# Predictors of adherence to prescribed exercise programs for older adults with medical or surgical indications for exercise: a systematic review

**DOI:** 10.1186/s13643-022-01966-9

**Published:** 2022-04-29

**Authors:** Julia F. Shaw, Sophie Pilon, Matthieu Vierula, Daniel I. McIsaac

**Affiliations:** 1 Ottawa Hospital Research Institute, Clinical Epidemiology Program, 1053 Carling Ave, Ottawa, ON K1Y 4E9 Canada; 2 School of Epidemiology & Public Health, University of Ottawa, 600 Peter Morand Crescent, Ottawa, ON K1G 5Z3 Canada; 3 Departments of Anesthesiology & Pain Medicine, University of Ottawa and The Ottawa Hospital, Ottawa, ON Canada

**Keywords:** Exercise, Systematic review, Prognosis, Evidence-based medicine

## Abstract

**Background and objectives:**

Prescribed exercise to treat medical conditions and to prepare for surgery is a promising intervention to prevent adverse health outcomes for older adults; however, adherence to exercise programs may be low. Our objective was to identify and grade the quality of predictors of adherence to prescribed exercise in older adults.

**Methods:**

Prospective observational and experimental studies were identified using a peer-reviewed search strategy applied to MEDLINE, EMBASE, Cochrane, and CINAHL from inception until October 6, 2020. Following an independent and duplicate review of titles, abstracts, and full texts, we included prospective studies with an average population age >65 years, where exercise was formally prescribed for a medical or surgical condition. We excluded studies where exercise was prescribed for a chronic musculoskeletal condition. Risk of bias was assessed using the Quality in Prognostic studies tool or Cochrane risk of bias tool, as appropriate. Predictors of adherence were identified and graded for quality using an adaptation of the Grading of Recommendations Assessment, Development, and Evaluation (GRADE) framework for predictor studies.

**Results:**

We included 19 observational studies and 4 randomized controlled trials (*n*=5785) Indications for exercise included cardiac (*n*=6), pulmonary rehabilitation (*n*=7), or other (*n*=10; surgical, medical, and neurologic). Of the 10 studies that reported adherence as the percent of prescribed sessions completed, average adherence was 80% (range 60–98%; standard deviation (SD) 11%). Of the 10 studies that reported adherence as a categorical threshold demarking adherent vs not adherent, average adherence was 57.5% (range 21–83%; SD 21%). Moderate-quality evidence suggested that positive predictors of adherence were self-efficacy and good self-rated mental health; negative predictors were depression (high quality) and distance from the exercise facility. Moderate-quality evidence suggested that comorbidity and age were not predictive of adherence.

**Conclusions:**

These findings can inform the design of future exercise programs as well as the identification of individuals who may require extra support to benefit from prescribed exercise.

**Systematic review registration:**

PROSPERO CRD42018108242

**Supplementary Information:**

The online version contains supplementary material available at 10.1186/s13643-022-01966-9.

## Background

Western populations are aging at a rapid rate; it is estimated that by 2050, adults over the age of 65 could account for up to 30% of our population [1]. The declining physical function that accompanies older age is associated with increased disability, institutionalization, and mortality [2]. Additionally, frailty, a multidimensional syndrome related to age- and disease-related deficits, increases in prevalence with age and results in vulnerability to stressors and adverse health outcomes [3, 4]. Therefore, a large proportion of older individuals facing physiologic stressors, such as surgery or chronic medical conditions, are at risk of suffering worse outcomes compared to those who are more physically fit.

Older individuals preparing for surgery or who have medical problems may benefit from interventions that target increasing their physical reserve to improve outcomes [5]. Exercise has been identified as a promising perioperative intervention to improve postoperative outcomes in vulnerable older adults having surgery [6], may reduce mortality after cardiac events [7], and is a key aspect in managing peripheral artery disease and pulmonary disease [8, 9]. While exercise shows encouraging results for the treatment and prevention of adverse health outcomes in older adults [6], participants must adhere to the prescribed program in order to benefit from the exercise intervention [10, 11]. However, it is well-documented that older adults’ adherence to prescribed exercise programs is low, especially in those with complex health conditions [12–16]. To support successful implementation of exercise programs for older adults, we must first identify what factors influence adherence to these programs to ensure that participants are willing and able to comply. To our knowledge, no studies have synthesized and graded the strength of evidence for patient- and program-level factors that predict exercise adherence.

To address this gap in the literature, our objective was to identify and grade the quality of predictors of adherence to prescribed exercise in older adults with either a medical or surgical indication (at any time point in their course of illness, i.e., as rehabilitation or prehabilitation). This systematic review will provide knowledge to inform current care and future research regarding the implementation and design of exercise programs for older adults with medical and surgical indications for exercise.

## Methods

### Design

This was a systematic review that followed best practice recommendations from the Cochrane Collaboration [17] and for systematic reviews of observational and prognostic studies [18, 19]. We pre-registered our protocol (PROSPERO 2018 CRD42018108242) and reported findings using the Preferred Reporting Items for Systematic reviews and Meta-Analyses (PRISMA) guidelines (see Additional file 1) [20]. All stages of the review were conducted using Distiller SR (Evidence Partners, Ottawa, Canada), a cloud-based systematic review platform.

### Search strategy

A search strategy was developed in consultation with an information specialist (see Supplementary Table S2; Additional file 2) and peer-reviewed [21]. Citations were extracted from MEDLINE, Embase, Cochrane, and CINAHL from inception until October 2020. Only English or French studies were included, as reviewers were only English- and French-speaking. All reference lists were reviewed to identify relevant studies that could have been missed by our database searches.

### Eligibility criteria

Studies were eligible for inclusion if the following criteria were met [1]: average age of participants ≥65 years [2]; participants had a medical or surgical condition as an indication for exercise [3]; participants were prescribed or recommended a formal exercise program, and [4] any predictors of exercise adherence were reported. Prior to beginning our review, we recognized that exercise programs for chronic musculoskeletal conditions (e.g., low back pain or chronic joint pain, or arthritis) *versus* other indications would be a primary source of heterogeneity. We also identified several syntheses of adherence in chronic musculoskeletal conditions that were already available [22–26]; therefore, we excluded studies where chronic musculoskeletal conditions were the indication for exercise. Study designs were limited to prospective observational or experimental studies to minimize the effects of misclassification bias and measurement error. Effect estimates predictive of adherence were limited to those that underwent multivariable adjustment to minimize confounding bias, as recommended by best practice guidelines [19]. This meant that we included [1]: adjusted associations between participant or program characteristics and adherence reported from prospective cohort studies or the experimental arm of randomized trials of prescribed exercise [2] or the effect estimate from a randomized trial if it estimated the effect of two different program features on adherence.

### Study selection and data extraction

Title and abstract screening was performed in duplicate (JFS, MV, SP); any studies reviewed as “yes” or “unsure” by either reviewer were advanced to full-text review. Agreement between both reviewers was required to exclude a study. Full-text articles were also assessed in duplicate (JFS, MV, SP) and reasons for exclusion at this stage were recorded and categorized (wrong age group, no exercise program, no predictors of adherence, no medical or surgical condition, wrong study design, and other). Disagreements between reviewers during full-text review were resolved by consensus through discussion with the senior author (DIM).

A unique data extraction form was created for this study. The form was piloted in a sample of 8 studies by two extractors (MV, SP), which were then reviewed with the senior author. Following piloting, data was extracted by one reviewer and independently reviewed and checked for accuracy by a second reviewer. Extracted data included publication details (author, year), study design, sample size, average age, medical/surgical condition indicating exercise, and whether frailty status was assessed. We also extracted characteristics at the exercise program level, including inpatient/outpatient, supervised/unsupervised, type of program (i.e., cardiac rehabilitation, pulmonary rehabilitation, or other), and effect estimates or p-values of predictors of adherence. Our primary outcome, program adherence, was recorded, including the definition used to quantify adherence and the overall adherence rate reported.

### Risk of bias assessment

Risk of bias was evaluated independently in duplicate (JFS, MV, SP), and disagreements were resolved through discussion with the senior author (DIM). Randomized controlled trials were assessed using the Cochrane risk of bias tool for randomized trials [17], while observational studies were assessed using the Quality in Prognostic Studies (QUIPS) tool [27].

### Synthesis of results and analysis

Our primary analysis was structured to support the Grading of Recommendations Assessment, Development and Evaluation (GRADE) adaptation for the prognostic factor research framework [28].

First, we categorized studies based on the indication for exercise (cardiac rehabilitation, pulmonary rehabilitation, and others). Next, prognostic factors were identified and categorized within themes (based on a consensus meeting within the investigative team). Where a prognostic factor was reported by two or more studies, the strength and quality of the association of the predictive factor with adherence was assigned using the GRADE prognostic factor framework. This process applies 8 criteria that can upgrade or downgrade the quality of evidence supporting a prognostic factor and allows for evidence of a review of prognostic factors to be efficiently summarized for end-users [28].

We also calculated descriptive statistics for the overall collection of included studies, as well as by indication for exercise. Overall adherence rates were calculated and averaged across all studies, as well as by exercise indication category. Adherence measures were separated based on the measurement on a continuous scale (i.e., percent of prescribed exercise completed) or as a binary measurement (i.e., categorical threshold demarking adherent vs not adherent).

Due to the heterogeneity of the included studies, meta-analysis and subgroup analyses were not performed.

## Results

### Study selection

**Fig. 1:**
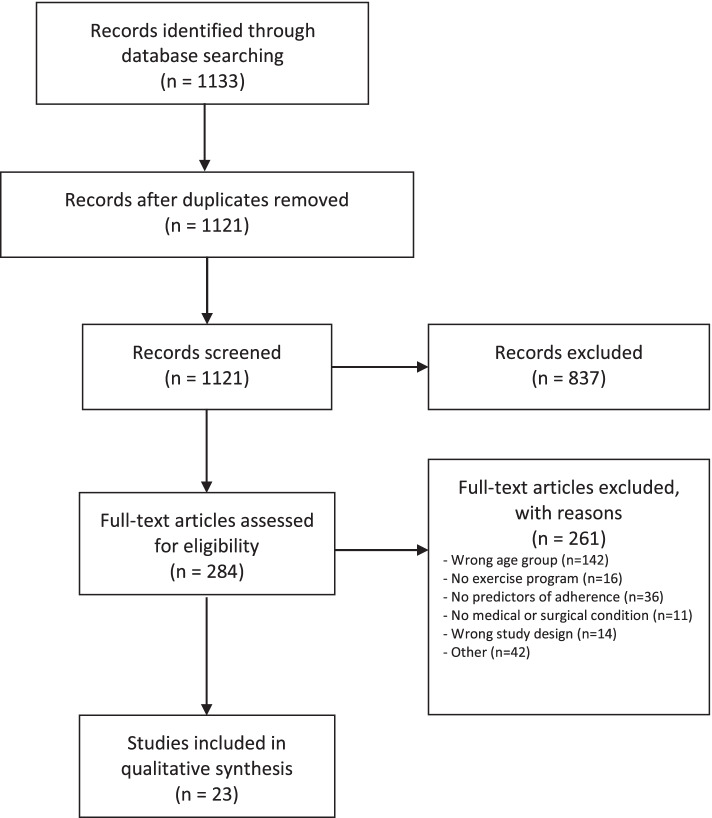
PRISMA flow diagram for study selection and inclusion

The search strategy identified 1133 records; 1121 remained after duplicates were removed. Following title and abstract screening, 284 full-text articles were assessed for eligibility and 23 were included. Study selection and reasons for exclusion are presented in Fig. 1, with reasons for exclusion at full-text review documented in see Supplementary Table S3 (Additional file 3).

### Study characteristics

Study characteristics are presented in Table 1. Nineteen observational studies and 4 randomized controlled trials were included. Countries of origin of studies included Australia, Canada, USA, UK, Ireland, Denmark, and Netherlands. A total of 5651 individuals were prescribed exercise across all studies (sample sizes ranged from 30–1218 participants) and the average age ranged from 66–79 years. Indications for exercise included cardiac rehabilitation (*n*=6), pulmonary rehabilitation (*n*=7), and other (*n*=10; including surgical, medical, and neurologic indications). Most (20/23 (87%)) exercise programs were supervised.

**Table 1:** Study characteristics

Author	Year	Design	Country	***N***	Average age	Medical/surgical indication	Exercise program	Overall Adherence	Adherence definition
Ades et al. [29]	1992	OBS	USA	226	70	MI or CABG	CR^a^	21%^c^	Entry rate into the CR
Aherne et al. [30]	2017	OBS	Ireland	98	69	PVD	Other^a^	19.5±24.9(mean)	Number of sessions attended
Brown et al. [31]	2016	OBS	USA	440	66	COPD	PR^a^	52%^c^	≥8 weeks completed
Casey et al. [32]	2008	OBS	USA	600	66	CVD	CR^a^	78%^c^	Sufficient completion by staff judgment
Covey et al. [33]	2014	RCT	USA	113	68	COPD	PR^a^	95%^b^	Percent of prescribed sessions completed
Cox et al. [34]	2013	OBS	Australia	85	68	Cognitive impairment	Other	78%^b^	Percent of prescribed sessions completed
Craike et al. [35]	2016	OBS	Australia	52	67	Prostate cancer	Other^a^	80%^b^	Percent of supervised component sessions attended
Fan et al. [36]	2008	OBS	USA	1218	67	COPD	PR^a^	79%^c^	Completion of all 10 prescribed sessions
Gallagher et al. [37]	2003	OBS	Australia	196	67	CVD	CR^a^	32%^c^	Completion of any CR program
Hogg et al. [38]	2012	OBS	UK	812	>65	COPD	PR^a^	54%^c^	≥50% attendance (rolling recruitment program) or ≥75% attendance (cohort recruitment program)
Jensen et al. [39]	2016	OBS	Denmark	50	69	Bladder cancer surgery	Other	66%^c^	≥75% program completion
Karssemeijer et al. [40]	2019	RCT	Netherlands	115	79	Dementia	Other^a^	85%^b^	Percent of prescribed sessions attended
Messer et al. [41]	2007	OBS	USA	164	66	Incontinence	Other^a^	68%^c^	Self-reported 2-3 sessions/week at 12 months follow-up
Mudge et al. [42]	2013	OBS	Australia	140	> 65	CVD, pulmonary disease	Other^a^	42%^c^	≥6 weeks attendance
Pakzad et al. [43]	2013	OBS	Canada	30	66	CVD	CR^a^	N/A	Number of sessions completed
Pandey et al. [44]	2017	RCT	Canada	40	67	Diabetes	Other^a^	69%^b^	Percent of prescribed minutes of exercise completed per month from self-report
Pickering et al. [45]	2013	OBS	UK	70	73	Parkinson's disease	Other^a^	79%^b^	Percent of prescribed repetitions completed
Rizk et al. [46]	2015	RCT	Canada	35	67	COPD	PR^a^	75%^b^	Percent of prescribed sessions attended
Selzler et al.^47h^	2016	OBS	Canada	64	69	COPD	PR^a^	81%^b^	Percent of prescribed sessions attended
Selzler et al.^48h^	2012	OBS	Canada	814	68	COPD	PR^a^	83%^c^	≥50% attendance
Tiedemann et al. [47]	2012	OBS	Australia	76	67	Stroke	Other^a^	60%^b^	Percent of prescribed sessions attended
Tooth et al. [48]	1993	OBS	Australia	30	66	MI	CR	93%^bf^, 87%^bg^	Percent of prescribed exercise completed
van Montfort et al. [49]	2016	OBS	Netherlands	409	66	PCI	CR^a^	25.6(mean score)	Self-reported general treatment adherence (Medical Outcomes Study questionnaire)

*CABG* coronary artery bypass graft, *COPD* chronic obstructive pulmonary disease, *CR* cardiac rehabilitation, *CVD* cardiovascular disease, *FEV1* forced expiratory volume in 1 second, *HADS* Hospital Anxiety and Depression Scale, *HDL* high density lipoprotein, *IMD* Index of Multiple Deprivation, *MI* myocardial infarction, *MRC* Medical Research Council, *OBS* observational, *PCI* primary coronary intervention, *PR* pulmonary rehabilitation, *PVD* peripheral vascular disease, *RCT* randomized controlled trial

^a^Supervised exercise program; ^b^adherence as a continuous percent, ^c^adherence as a binary threshold; ^d^% participation; ^e^% completion; ^f^% duration; ^g^% frequency, ^h^participants in Selzler et al. studies are unique.

### Adherence to prescribed exercise rates

Exercise adherence was measured as a continuous percent of prescribed exercise sessions attended or completed in 10 studies, as a binary outcome with a specified cut-off (adherent vs not adherent) in 10 studies, as the number of sessions completed in 2 studies, and as a score of self-reported adherence in 1 study. Definitions for continuous measures of adherence included the percent of prescribed sessions completed (*n*=3) or attended (*n*=5), and of repetitions/minutes completed (*n*=2). Definitions for binary measures of adherence included entry into the program (*n*=1), a threshold number of weeks/sessions of program completed (*n*=3) or attended (*n*=3), sufficient completion by staff judgment (*n*=1), completion of any CR program (*n*=1), and continued self-reported adherence at 12 months (*n*=1). Of the 10 studies reporting adherence as a continuous percent, average overall adherence was 79% (range 60–95%; standard deviation (SD) 10%). Percent frequency value from Tooth et al. was used for combined measure. Of the 10 studies reporting adherence as a categorical threshold demarking adherent vs not adherent, average adherence was 58% (range 21–83%; SD 21%). Mean overall adherence for cardiac rehabilitation was 87% (continuous rate; *n*=1) and 44% (SD 30%; binary threshold; *n*=3). Mean overall adherence for pulmonary rehabilitation was 84% (SD 10%; continuous rate; *n*=3) and 67% (SD16%; binary threshold; *n*=4). Mean overall adherence for other indications was 75% (SD 9%; continuous rate; *n*=6) and 59% (SD14%; binary threshold; *n*=3). However, a lack of variance measures around adherence estimates limited our ability to perform formal comparative meta-analysis or meta-regression.

### Predictors of exercise adherence

Predictors of exercise adherence were grouped into the following clusters: demographic, psychological, program-related, medical condition severity, comorbidities, and others. Demographic factors were evaluated by 13 studies [32, 35–39, 42, 43, 45, 48–51] (Table 2), psychological factors by 14 studies [29, 31, 32, 34–38, 41, 43, 45, 48–51] (Table 3), program-related factors by 7 studies [29, 33, 36, 40, 42, 44, 46], medical condition severity by 11 studies [31, 35–38, 45, 47–51], comorbidities by 8 studies [31, 32, 34, 39, 43, 48, 50, 51] and other predictors by 5 studies [29, 38, 42, 47, 48]. Similar factors were investigated between exercise indications (see Supplementary Tables S4, S5, S6; Additional files 4, 5, 6), however more functional measures to quantify lung function were used as potential predictors of adherence among pulmonary rehabilitation studies, while more disease characteristics and history measures were used among cardiac rehabilitation studies.

**Table 2:** Demographic predictors of exercise adherence

Study	Predictors	Direction	Theme
Casey et al. (2008) [32]	Age (years)	+	Age
	Employed (vs not employed/retired)	0	Employment
	Gender (male vs female)	0	Sex
Craike et al. (2016) [35]	Highest level of education (less than university degree vs university degree or higher)	0	Education
Fan et al. (2008) [36]	Age (per 1 year change)	0	Age
	Female gender	0	Sex
	Education reference: < high school		Education
	High school	+	
	Some college	+	
	> College	+	
Gallagher et al. (2003) [37]	Unemployed or retired (vs employed)	−	Employment
	Age > 70 (vs 55–70)	−	Age
Hogg et al. (2012) [38]	Deprivation quintile (IMD score) reference: IMD 6.86–28.1		Social status
	IMD 28.11–35.02	0	
	IMD 35.03–39.57	0	
	IMD 39.58–43.85	−	
	IMD 43.86–60.41	−	
Jensen et al. (2016) [39]	Gender (women vs men)	0	Sex
	Age (<70 vs ≥70)	0	Age
Mudge et al. (2013) [42]	Retired from workforce (vs “working” and “not working”)	+	Employment
	Age <65 vs 65+	0	Age
	Sex (male vs female)	0	Sex
	Living alone vs living with family/others	0	Living status
Pakzad et al. (2013) [43]	Identity	0	
Pickering et al. (2013) [45]	Gender (male vs female)	0	Sex
	Living status (alone vs partner vs family/friends vs other)	0	Living status
	Age multiplicative decrease per 10 years	−	Age
Selzler et al. (2016) [51]	Age (years)	0	Age
Selzler et al. (2012) [50]	Age (years)	+	Age
Tooth et al. (1992) [48]	Scale of status and prestige (high score = lower social standing)	−	Social status
	Age (years)	0	Age
	Education (years)	0	Education
van Montfort et al. (2016) [49]	Female sex (vs male)	0	Sex
	Age (years)	−	Age

*IMD* Index of Multiple Deprivation (0, the least deprived, to 86, the most deprived); scale of status and prestige (1 to 7, where 1 represents occupations of the highest social standing); + = significant positive effect; 0 = no significant effect; **−** = significant negative effect

**Table 3:** Psychological predictors of exercise adherence

Study	Predictors	Direction	Theme
Ades et al. (1992) [29]	Presence of depression before hospitalization	−	Depression
Brown et al. (2016) [31]	Beck Depression Index	0	Depression
Casey et al. (2008) [32]	Beck Depression Index (high scores, more depressed)	−	Depression
Cox et al. (2013) [34]	Baseline self-efficacy (higher)	+	Self-efficacy
Craike et al. (2016) [35]	Role functioning (higher)	+	
	Sexual activity	0	
Fan et al. (2008) [36]	State-Trait Anxiety Index ≥ 36	−	Anxiety
	Beck Depression Index ≥ 5	−	Depression
Gallagher et al. (2003) [37]	Perceived control	0	Control
	Personal stressful event	-	
Hogg et al. (2012) [38]	Hospital Anxiety and Depression Score "Not depressed" 0-7	Reference	Depression
	“Risk of depression” 8-10	0	
	“Depressed” 11	−	
Messer et al. (2007) [41]	Task self-efficacy summary scores (higher)	+	Self-efficacy
	Regulatory self-efficacy summary scores (higher)	+	
	Knowledge self-efficacy	0	
van Montfort et al. (2016) [49]	Optimism (Revised Life Orientation Test)	+	
	Depression (Patient Health Questionnaire (PHQ-9))	0	Depression
	Anxiety (Generalized Anxiety Disorder (GAD-7) scale)	−	Anxiety
Pakzad et al. (2013) [43]	State-Trait Anxiety Index (higher)	+	Anxiety
	Consequences	0	
	Chronology (acute/chronic)	0	
	Treatment control	0	
	Personal control	0	
Pickering et al. (2013) [45]	EQ-5D state of health thermometer	+	
	EQ-5D No pain/discomfort	Reference	
	EQ-5D Moderate pain/discomfort	0	
	EQ-5D Extreme pain/discomfort	−	
	EQ-5D Not anxious/depressed	Reference	Anxiety, Depression
	EQ-5D Moderate anxious/depressed	−	
	EQ-5D Extreme anxious/depressed	−	
	Mental health problem (self-reported)	−	Mental health
Selzler et al. (2012) [50]	Social functioning (36-Item Short Form Survey)	+	
	Mental health (36-Item Short Form Survey)	+	Mental health
	Role emotional (36-Item Short Form Survey)	+	
Selzler et al. (2016) [51]	Task self-efficacy	+	Self-efficacy
	Coping self-efficacy	0	
	Scheduling self-efficacy	0	
Tooth et al. (1992) [48]	Expectations (higher)	+	
	Psychological status (profile of mood states score)	0	

+ = significant positive effect; 0 = no significant effect; **-** = significant negative effect

### GRADE recommendations

Prognostic factors, categorized by themes, reported by at least 2 observational studies were assessed using the GRADE framework (Table 4). Overall, studies reported a small to negligible effect of most predictors. However, lower socioeconomic status (SES), the presence of depression, and farther distance from exercise facility predicted lower adherence, while higher self-efficacy and good mental health predicted higher adherence. Additionally, a dose-response effect was only observed for the predictors of depression and higher self-efficacy.

**Table 4:** Grading of recommendation assessment, development, and evaluation

Predictors	Participants	Studies	+	0	−	Phase	Limitations	Inconsistency	Indirectness	Imprecision	Publication bias	↑ effect size	Dose effect	Quality	Effect
***Demographic***															
Age (older)	3591	10	2	5	3	2	✓	X	✓	✓	✓	∅	∅	M	~
Sex (male)	2487	6		6		2	✓	✓	✓	✓	✓	∅	∅	H	~
Employed	936	3	1	1	1	2	✓	X	✓	✓	X	∅	∅	L	~
More education	1300	3	1	2		2	✓	X	✓	X	X	∅	∅	VL	~
Living alone	210	2		2		2	✓	X	✓	✓	X	∅	∅	L	~
Lower SES	842	2			2	2	✓	✓	✓	X	X	∅	∅	L	↓
***Psychological***															
Anxiety	1727	4	1		3	2	✓	X	✓	✓	X	∅	∅	L	~
Depression	3775	7		2	5	2	✓	✓	✓	✓	✓	∅	Δ	H	↓
Higher self-efficacy	313	3	3			2	✓	✓	✓	X	X	∅	Δ	M	↑
Higher control	226	2		2		2	✓	✓	✓	X	X	∅	∅	L	~
Good mental health	884	2	2			2	✓	✓	✓	✓	X	∅	∅	M	↑
***Comorbidities***															
High BMI	1848	3		3		2	✓	✓	✓	✓	X	∅	∅	M	~
Smoker	1446	5	1	2	2	2	✓	X	✓	✓	X	∅	∅	L	~
High cholesterol	158	3	1	2		2	✓	X	✓	X	X	∅	∅	VL	~
Hypertension	128	2		2		2	✓	✓	✓	X	X	∅	∅	L	~
Higher CCI	1268	2		2		2	✓	✓	✓	✓	X	∅	∅	M	~
***Condition severity***															
Better respiratory function	878	2	1	1		2	✓	X	✓	✓	X	∅	∅	L	~
Higher FEV1	1658	2	1	1		2	✓	X	✓	✓	X	∅	∅	L	~
***Program***															
Farther distance	1444	2			2	2	✓	✓	✓	✓	X	∅	∅	M	↓
Continuous exercise (vs intermittent)	75	2^a^	1		1	2	✓	X	✓	✓	X	∅	∅	M	~
***Other***															
Exercise history	160	2	1	1		2	✓	X	✓	X	X	∅	∅	VL	~

+ = number of studies with a significant positive effect; 0 = number of studies with no significant effect; − = number of studies with a significant negative effect; ✓ = no serious limitations; ^a^randomized controlled trials; *X* serious limitations, Δ= present, ∅ not present, *M* moderate, *H* high, *L* low, *VL* very low

#### Demographics

Demographic predictors included age, sex or gender, employment, education, living situation, and social status. Lower socioeconomic status was the only demographic predictor to have an effect on adherence. Low-quality evidence suggested that lower socioeconomic status predicted lower adherence. High-quality evidence suggested that sex did not predict adherence and moderate-quality evidence suggested that age did not predict adherence. Low, low, and very low-quality evidence, respectively, suggested that employment status, living status, and education were also not predictive of adherence.

#### Psychological factors

Psychological predictors included anxiety, depression, self-efficacy, perception of control, and self-rated mental health. High-quality evidence supported a negative association between the presence of depression and adherence. Moderate-quality evidence suggested that individuals who had good self-rated mental health and good self-efficacy had higher adherence. Low-quality evidence suggested that the presence of anxiety and perception of control did not predict adherence.

#### Comorbidities

Identified comorbidities reported as predictors of exercise adherence were Body Mass Index (BMI), smoking status, hypercholesterolemia, hypertension, and Charleston Comorbidity Index (CCI). None of these were predictive of exercise adherence, which was supported by moderate-quality evidence for BMI and CCI, low-quality evidence for smoking status and hypertension, and very low-quality evidence for hypercholesterolemia. Frailty was measured and reported in 1 study [40], but was not assessed as a predictor of exercise program adherence.

#### Medical condition severity

Measures of respiratory disease severity (better respiratory function and higher FEV1) were not found to be predictive of adherence, supported by low-quality evidence.

#### Program factors

Program-level predictors included farther distance from the exercise facility and continuous vs intermittent exercise. The type of exercise program (continuous vs intermittent) was evaluated by two randomized controlled trials, which suggested no association with adherence. Although randomized trials are considered to provide high-quality evidence, we downgraded the quality of evidence to moderate-quality, given that trial findings were contradictory (one trial reported better adherence to interval exercise, one reported better adherence with continuous exercise). Moderate-quality evidence suggested that living a farther distance from the exercise facility decreased adherence.

#### Other

Low-quality evidence suggests that a history of exercise participation is not predictive of exercise adherence.

### Risk of bias within studies

Nine observational studies were deemed to be at low risk of bias and 10 were at moderate risk of bias; no studies were at high risk of bias (Supplementary Table S7). Importantly, prognostic factor measurement and study confounding components of the tool scored low risk of bias across all studies. All four randomized trials were assessed as high risk of bias due to lack of blinding, however, this is recognizably difficult in exercise interventions (Supplementary Table S8). One trial had a high risk of bias in the blinding of outcome assessment domain; all other domains were low or unclear risk of bias.

## Discussion

In this systematic review of predictors of exercise adherence in older adults with medical or surgical indications for prescribed exercise, we found that positive predictors of adherence, supported by moderate-quality evidence, were higher self-efficacy and good self-rated mental health. Negative predictors included depression (high-quality) and distance from the exercise facility (moderate quality). Interestingly, comorbidity status, sex, and age did not appear to be predictive of adherence (supported by moderate- to high-quality evidence), as none suggested a directional association. We also found that having a history of exercise participation is not a predictor of exercise program adherence. This was a surprising finding because a common assumption is that people who have exercised previously will be more adherent to a program in the future. However, this finding was supported by low-quality evidence and therefore needs further investigation. As prescribed exercise programs are less likely to be effective without high levels of adherence, these findings provide important insights into current practice and future research. However, it should be noted that while most demographic, comorbidity, and psychological predictors were evaluated in all exercise indications with similar results, self-efficacy and self-rated mental health were not evaluated in any cardiac rehabilitation studies. Additionally, distance from the exercise facility was only evaluated in cardiac and pulmonary rehabilitation studies. The small number of identified predictors with at least moderate-quality evidence and the sparse data available for many predictors suggest that future research is needed in a variety of medical and surgical indications to better understand and predict exercise adherence in older adults.

Previous reviews have estimated exercise adherence rates in a variety of populations, typically reporting similar adherence rates as those identified in our study. For example, Bullard et al. [52] reported a pooled adherence rate of 77% (95% CI 68–84%) across 30 studies of adults with cancer, cardiovascular disease or diabetes. However, few studies have evaluated what patient- and program-factors predict adherence, and to our knowledge, none have evaluated the strength of this evidence using a standard framework such as GRADE. Similar to our findings, Morgan et al. [53] identified program location as a barrier to participation and adherence, while Sheill et al. [54] found that difficulties traveling to exercise locations were a substantial barrier for individuals with advanced cancer. We found no evidence that the type of exercise program (i.e., interval vs continuous exercise) was predictive of adherence, which is consistent with recommendations that the act of engaging in exercise is likely of greater importance than the specific type of exercise performed [52, 54].

Some authors have advocated the identification of participant-level ‘red flags’ to adherence as a way to personalize exercise program design and support [52]. However, this approach requires a thorough understanding of what participant characteristics may act as red flags. At the participant level, consistent findings from our study and from others suggest that aspects of mental health are likely key predictors of adherence. The presence of depression was a strong negative predictor of adherence and the only predictor supported by high-quality evidence, while good self-rated mental health was a strong positive predictor of adherence, supported by moderate-quality evidence. In practice, identification of negative predictors, with a particular focus on mental health, could allow for increased personalization and targeting of support. For example, mental health aspects could be targeted by providing social support systems, as tangible and emotional support have been shown to be associated with lower depression among older adults [55–57]. The other negative predictor of exercise adherence in this study, distance from the exercise facility, could be targeted by developing and providing home-based exercise programs, which would eliminate this barrier altogether. Self-efficacy has previously been reported as a predictor of adherence in a systematic review of home-based physiotherapy [58], which is consistent with our findings and aligns with other systematic reviews that have found one’s intentions to engage in health-changing behaviors to be strongly predictive of adherence [59]. In practice, self-efficacy could be increased in patients by targeting sources of self-efficacy, such as verbal persuasion by providing positive feedback, or mastery experiences by telling patients to reflect on a time when they successfully acted upon an intention [60].

Moreover, obesity and multimorbidity were the only comorbidities with at least moderate-quality evidence as predictors. Many comorbidities were not assessed, and the impact of frailty was not reported in any studies, suggesting a need for future research to investigate the role of frailty as a potential predictor of exercise adherence. Finally, absent from the literature and related reviews is the consideration that program factors may interact with participant factors when predicting adherence. Although we were unable to identify any evidence of this phenomenon in our review, future evaluation is likely warranted to understand how participant-level red flags such as poor mental health may be modified by specifically targeted aspects of program design. Such efforts could lead to better personalization and potentially higher adherence in individuals at risk of poor participation.

### Strengths and limitations

Our study’s findings should be considered in the context of its strengths and limitations. First, we conducted our review according to best-practice methodologies, which included protocol pre-registration, peer-review of our search strategy, review of multiple databases, a focus on adjusted estimates and contextualization of our findings within the GRADE strength of evidence framework. Furthermore, our results are based on identified studies that were generally at low or moderate risk of bias (apart from blinding issues in randomized trials, which is typical of exercise studies). However, despite pre-specifying a defined population of interest, included studies represented a somewhat heterogenous group of participants who engaged in exercise for cardiovascular, pulmonary, and other indications. We were also unable to adequately identify homogenous data to support quantitative meta-analyses. This may, in part, reflect the number of largely unvalidated measures used to define exercise adherence in clinical research [61]. Accordingly, we classified our studies based on whether adherence was measured using a continuous or binary definition; however, this may not have completely captured the heterogeneity in underlying adherence measures. Additionally, included studies were based in countries in Europe, North America, and Australia; therefore, findings might be limited in their generalisability to low- and middle-income countries. Finally, our search was restricted to English or French articles and we did not search unpublished data sources.

## Conclusions

Design of prescribed exercise programs for older adults requires an understanding of how program and participant characteristics impact exercise adherence. Based on the GRADE Framework for prognostic research, mental health factors appear to be the most important patient-level predictors, while a farther distance from the exercise facility was the only clear program-related factor predicting adherence. These findings can help to inform the design of current programs and personalization of support for participants.

## Supplementary Information

**Additional file 1.**

**Additional file 2: Supplementary Table S2.** Search Strategy.

**Additional file 3: Supplementary Table S3.** Full Text Review Reasons for Exclusion.

**Additional file 4: Supplementary Table S4.** Predictors of Exercise Adherence for Cardiac Rehabilitation.

**Additional file 5: Supplementary Table S5.** Predictors of Exercise Adherence for Pulmonary Rehabilitation.

**Additional file 6: Supplementary Table S6.** Predictors of Exercise Adherence for Other Exercise Programs.

**Additional file 7: Supplementary Table S7.** Risk of Bias Assessments for Observational Studies (QUIPS tool).

**Additional file 8: Supplementary Table S8.** Risk of Bias Assessments for Randomized Controlled Trials (Cochrane Risk of Bias tool).

## Data Availability

Not applicable

## Abbreviations

QUIPS: Quality in Prognostic Studies
GRADE: Grading of Recommendations Assessment, Development and Evaluation
BMI: Body mass index
CCI: Charleston Comorbidity Index
