# Supplementary material for: Predictors of adherence to prescribed exercise programs for older adults with medical or surgical indications for exercise: a systematic review
Source: Syst Rev. 2022 Apr 29;11:80. doi: 10.1186/s13643-022-01966-9 (PMC9052492; doi:10.1186/s13643-022-01966-9)
Supplement: Supplementary file 2 — Additional file 2: Supplementary Table S2. Search Strategy. [file 13643_2022_1966_MOESM2_ESM.docx]

**Supplementary Table S2. Search Strategy**

Database: Embase Classic+Embase, Ovid MEDLINE(R) Epub Ahead of Print, In-Process & Other Non-Indexed Citations, Ovid MEDLINE(R) Daily and Ovid MEDLINE(R)

Search Strategy:

--------------------------------------------------------------------------------

1 exp *exercise/ or exercise/ (420369)

2 exercise physiology/ (890)

3 exp kinesiotherapy/ (68606)

4 heart rehabilitation/ or rehabilitation/ (110657)

5 exp physiotherapy/ (81699)

6 (exercis* or physical therap* or physiotherap* or rehabilitat* or prehabilitat*).tw. (999646)

7 or/1-6 (1229851)

8 patient compliance/ (176050)

9 (compliance or adherence).tw. (479012)

10 8 or 9 (566383)

11 7 and 10 (34680)

12 (validate or validation or predict*).tw. or *prediction/ (3464520)

13 11 and 12 (3819)

14 aged/ (5622860)

15 frail elderly/ (18419)

16 (aged adj2 (6# or 7# or 8# or 9#)).tw. (284484)

17 (frail* or fragil*).tw. (122599)

18 or/14-17 (5814796)

19 13 and 18 (1211)

20 exp physical disease/ (19460657)

21 (cancer or neoplasm* or carcinoma or malignan* or tumor* or tumour*).tw. (6702399)

22 (copd or Chronic Obstructive Pulmonary Disease*).tw. (142119)

23 (medical* adj2 ill*).tw. (15951)

24 20 or 21 or 22 or 23 (22850482)

25 19 and 24 (578)

**26 25 use emczd (475) Embase**

27 Exercise/ph or exercise/ (350326)

28 exp Exercise Therapy/ (112539)

29 Cardiac Rehabilitation/ or Rehabilitation/ (112103)

30 exp Physical Therapy Modalities/ (221855)

31 (

32 27 or 28 or 29 or 30 or 31 (1299168)

33 Patient Compliance/ (176050)

34 (compliance or adherence).tw,kw. (484828)

35 33 or 34 (571213)

36 32 and 35 (36273)

37 predict*.tw. (3068950)

38 (validate or validation).tw. (534190)

39 37 or 38 (3464166)

40 36 and 39 (3927)

41 exp Aged/ (5665749)

42 (aged adj2 (6# or 7# or 8# or 9#)).tw. (284484)

43 (elderly or senior* or geriatric or older person* or older people or older adult* or older women or older men or older individual*).tw,kw. (862200)

44 (frail* or fragil*).tw,kw. (125026)

45 41 or 42 or 43 or 44 (6118563)

46 40 and 45 (1369)

47 exp “diseases (non mesh)”/ or disease/ (14348050)

48 (cancer or neoplasm* or carcinoma or malignan* or tumor* or tumour*).tw,kw. (6796544)

49 (copd or Chronic Obstructive Pulmonary Disease*).tw,kw. (144923)

50 (medical* adj2 ill*).tw. (15951)

51 47 or 48 or 49 or 50 (18853713)

52 46 and 51 (640)

**53 52 use ppez (521) Medline**

54 26 or 53 (996)

**55 remove duplicates from 54 (681)**

**56 55 use ppez (473) Medline**

**57 55 use emczd (208) Embase**
