# Supplementary material for: Predictors of adherence to prescribed exercise programs for older adults with medical or surgical indications for exercise: a systematic review
Source: Syst Rev. 2022 Apr 29;11:80. doi: 10.1186/s13643-022-01966-9 (PMC9052492; doi:10.1186/s13643-022-01966-9)
Supplement: Supplementary file 3 — Additional file 3: Supplementary Table S3. Full Text Review Reasons for Exclusion. [file 13643_2022_1966_MOESM3_ESM.docx]

**Supplementary Table S3. Full Text Review Reasons for Exclusion**

| **Citation** | **No program or prescription** | **No predictors** | **Age < 65** | **No medical indication** | **Retrospective** | **Other** |
| --- | --- | --- | --- | --- | --- | --- |
| Yelena Tarasenko,Chen Chen,Nancy Schoenberg. Self-Reported Physical Activity Levels of Older Cancer Survivors: Results from the 2014 National Health Interview Survey. Journal of the American Geriatrics Society. 2017///. 65:e39 | Y |  |  |  |  |  |
| Andrew S. Layne,Fang Chi Hsu,Steven N. Blair,Shyh Huei Chen,Jennifer Dungan,Roger A. Fielding,Nancy W. Glynn,Alexandra M. Hajduk,Abby C. King,Todd M. Manini,Anthony P. Marsh,Marco Pahor,Christine A. Pellegrini,Thomas W. Buford,Study LIFE. Predictors of Change in Physical Function in Older Adults in Response to Long-Term, Structured Physical Activity: The LIFE Study. Archives of physical medicine and rehabilitation. 2017///. 98:11 |  |  |  | Y |  |  |
| A. Soto-Varela,A. Faraldo-Garcia,M. Del-Rio-Valeiras,M. Rossi-Izquierdo,I. Vaamonde-Sanchez-Andrade,P. Gayoso-Diz,A. Lirola-Delgado,S. Santos-Perez. Adherence of older people with instability in vestibular rehabilitation programmes: prediction criteria. The Journal of laryngology and otology. 2017///. 131:232 |  |  |  |  |  | No multivariable |
| J. Taylor,S.E. Keating,M.D. Leveritt,D.J. Holland,S.R. Gomersall,J.S. Coombes. Study protocol for the FITR Heart Study: Feasibility, safety, adherence, and efficacy of high intensity interval training in a hospital-initiated rehabilitation program for coronary heart disease. Contemporary Clinical Trials Communications. 2017///. 8:181 |  |  |  |  |  | Study protocol |
| H. Rafique,G. Worley,C. Anele,A. Askari,O. Faiz. The use of prehabilitation in those undergoing colorectal surgery: A systematic review. Colorectal Disease. 2017///. 19:68 |  |  |  |  |  | Review |
| A.C. Santos,J.M. Ferro. The impact of anger in adherence to treatment and beliefs about disease 1 year after stroke. Journal of Neurology. 2017///. 264:1929 | Y |  |  |  |  |  |
| J.P. Singer,A. Soong,A. Bruun,S. Hays,J. Kukreja,A. Bracha,G. Chin,P.J. Wolters,M. Peters,C.M. Garvey. "pre-habilitation" of frail candidates for lung transplantation using a mobile health enabled home-based intervention is feasible and safe. American Journal of Respiratory and Critical Care Medicine. 2017///. 195:#pages# |  |  |  |  |  | Abstract only only |
| M.E. Taylor,S.R. Lord,H. Brodaty,S.E. Kurrle,S. Hamilton,E. Ramsay,L. Webster,N.L. Payne,J.C.T. Close. A home-based, carer-enhanced exercise program improves balance and falls efficacy in community-dwelling older people with dementia. International Psychogeriatrics. 2017///. 29:81 |  | Y |  |  |  |  |
| Karl Josef Franke,Ulrike Domanski,Maik Schroeder,Volker Jansen,Frank Artmann,Uwe Weber,Rainer Ettler,Georg Nilius. Telemonitoring of home exercise cycle training in patients with COPD. International journal of chronic obstructive pulmonary disease. 2016///. 11:2821 |  |  | Y |  |  |  |
| Soon Yeng Soo Hoo,Robyn Gallagher,Doug Elliott. Predictors of cardiac rehabilitation attendance following primary percutaneous coronary intervention for ST-elevation myocardial infarction in Australia. Nursing & health sciences. 2016///. 18:230 |  |  | Y |  |  |  |
| Yetsa A. Tuakli-Wosornu,Faith Selzer,Elena Losina,Jeffrey N. Katz. Predictors of Exercise Adherence in Patients With Meniscal Tear and Osteoarthritis. Archives of physical medicine and rehabilitation. 2016///. 97:1945 |  |  | Y |  |  |  |
| Ida Svege,Linda Fernandes,Lars Nordsletten,Inger Holm,May Arna Risberg. Long-Term Effect of Exercise Therapy and Patient Education on Impairments and Activity Limitations in People With Hip Osteoarthritis: Secondary Outcome Analysis of a Randomized Clinical Trial. Physical therapy. 2016///. 96:818 |  |  | Y |  |  |  |
| Pataraporn Kheawwan,Waraporn Chaiyawat,Yupin Aungsuroch,Yow Wu Bill Wu. Patient Readiness to Exercise After Cardiac Surgery: Development of the Readiness to Change Exercise Questionnaire. The Journal of cardiovascular nursing. 2016///. 31:186 |  |  | Y |  |  |  |
| W. Swardfager,P. Yang,N. Herrmann,K.L. Lanctot,B.R. Shah,A. Kiss,P.I. Oh. Depressive symptoms predict non-completion of a structured exercise intervention for people with Type 2 diabetes. Diabetic medicine : a journal of the British Diabetic Association. 2016///. 33:529 |  |  | Y |  |  |  |
| A. Ruano-Ravina,C. Pena-Gil,E. Abu-Assi,S. Raposeiras,Hof A. van 't,E. Meindersma,E.I. Bossano Prescott,J.R. Gonzalez-Juanatey. Participation and adherence to cardiac rehabilitation programs. A systematic review. International Journal of Cardiology. 2016///. 223:436 |  |  |  |  |  | Review |
| J.G. Quicke,N.E. Foster,R.O. Ogollah,P.R. Croft,M.A. Holden. Can attitudes and beliefs about exercise predict future physical activity level in older adults with knee pain?. Osteoarthritis and Cartilage. 2016///. 24:S488 |  |  |  |  |  | Abstract only only |
| Laura Q. Rogers,Amanda Fogleman,Steven Verhulst,Mudita Bhugra,Krishna Rao,James Malone,Randall Robbs,K.Thomas Robbins. Refining Measurement of Social Cognitive Theory Factors Associated with Exercise Adherence in Head and Neck Cancer Patients. Journal of psychosocial oncology. 2015///. 33:467 |  |  | Y |  |  |  |
| Saioa Chamosa,Jose A. Alarcon,Miren Dorronsoro,Francisco J. Madruga,Javier Barrera,Xabier Arrazola,Patxi de la Cuesta,Maria Eugenia Alkiza,Jose M. Begiristain,Inaki Carrera,Jesus M. San Vicente. Predictors of Enrollment in Cardiac Rehabilitation Programs in Spain. Journal of Cardiopulmonary Rehabilitation and Prevention. 2015///. 35:255 |  | Y | Y |  |  |  |
| Stefano Urbinati,Zoran Olivari,Lucio Gonzini,Stefano Savonitto,Rosario Farina,Maurizio Del Pinto,Alberto Valbusa,Giuseppe Fantini,Alessandra Mazzoni,Aldo P. Maggioni,BLITZ-4 Investigators. Secondary prevention after acute myocardial infarction: drug adherence, treatment goals, and predictors of health lifestyle habits. The BLITZ-4 Registry. European Journal of Preventive Cardiology. 2015///. 22:1548 | Y | Y |  |  |  |  |
| Rebecca N. Adams,Catherine E. Mosher,Cindy K. Blair,Denise C. Snyder,Richard Sloane,Wendy Demark-Wahnefried. Cancer survivors' uptake and adherence in diet and exercise intervention trials: an integrative data analysis. Cancer. 2015///. 121:77 |  |  |  |  |  | Review |
| E. Aartolahti,A.-M. Tolppanen,E. Lonnroos,S. Hartikainen,A. Hakkinen. Health condition and physical function as predictors of adherence in long-term strength and balance training among community-dwelling older adults. Archives of Gerontology and Geriatrics. 2015///. 61:452 |  |  |  | Y |  |  |
| L. Loew,L. Brosseau,G. Wells,Angelis G. De. Relevant determinants influencing walking adherence among older individuals with knee osteoarthritis: Participant exercise preference (PEP) pilot randomised clinical trial. Journal of Rheumatology. 2015///. 42:1312 |  |  |  |  |  | Abstract only only |
| E. Oosting,T. Hoogeboom,S. Appelman,J. Dronkers,Meeteren N. Van. Feasibility of an intensive therapeutic exercise program for frail elderly prior to total hip arthroplasty: Two randomized pilot studies. Physiotherapy (United Kingdom). 2015///. 101:eS1150 |  |  |  |  |  | Abstract only Only |
| R. Ayiesah,J.H. Leonard,C.Y. Chong. Development and validation of non-adherence to pulmonary rehabilitation questionnaire: a clinical tool for patients with chronic obstructive pulmonary diseases. La Clinica terapeutica. 2014///. 165:123 |  |  |  |  |  | Spanish |
| Mohamad Hasnan Ahmad,Suzana Shahar,Nur Islami Mohd Fahmi Teng,Zahara Abdul Manaf,Noor Ibrahim Mohd Sakian,Baharudin Omar. Applying theory of planned behavior to predict exercise maintenance in sarcopenic elderly. Clinical interventions in aging. 2014///. 9:1551 |  |  |  | Y |  |  |
| Angele McGrady,Robert Burkes,Dalynn Badenhop,Ron McGinnis. Effects of a brief intervention on retention of patients in a cardiac rehabilitation program. Applied psychophysiology and biofeedback. 2014///. 39:163 |  |  | Y |  |  |  |
| Monika E. Slovinec D'Angelo,Luc G. Pelletier,Robert D. Reid,Veronika Huta. The roles of self-efficacy and motivation in the prediction of short- and long-term adherence to exercise among patients with coronary heart disease. Health psychology : official journal of the Division of Health Psychology, American Psychological Association. 2014///. 33:1344 |  |  | Y |  |  |  |
| Andrew M. Busch,Lori A.J. Scott-Sheldon,Jacqueline Pierce,Elizabeth A. Chattillion,Karlene Cunningham,Maria L. Buckley,Jeffrey M. Mazer,Cerissa L. Blaney,Michael P. Carey. Depressed mood predicts pulmonary rehabilitation completion among women, but not men. Respiratory medicine. 2014///. 108:1007 |  |  |  |  | Y |  |
| Emmylou Beekman,Ilse Mesters,Erik J.M. Hendriks,Jean W.M. Muris,Geertjan Wesseling,Silvia M.A.A. Evers,Guus M. Asijee,Annemieke Fastenau,Hannah N. Hoffenkamp,Rik Gosselink,Onno C.P. van Schayck,Rob A. de Bie. Exacerbations in patients with chronic obstructive pulmonary disease receiving physical therapy: a cohort-nested randomised controlled trial. BMC pulmonary medicine. 2014///. 14:71 |  |  |  |  |  | No multivariable |
| Susan Cassidy,Sue Turnbull,Maria Gardani,Kim Kirkwood. Attendance at pulmonary rehabilitation classes: an exploration of demographic, physiological and psychological factors that predict completion of treatment. Chronic respiratory disease. 2014///. 11:95 |  |  |  |  | Y |  |
| Kathryn A. Murray,David J. Murphy,Sarah Jane Clements,Adrian Brown,Susan B. Connolly. Comparison of uptake and predictors of adherence in primary and secondary prevention of cardiovascular disease in a community-based cardiovascular prevention programme (MyAction Westminster). Journal of public health (Oxford, England). 2014///. 36:644 |  |  | Y |  |  |  |
| Bradley James Wright,Nicholas Justin Galtieri,Michelle Fell. Non-adherence to prescribed home rehabilitation exercises for musculoskeletal injuries: the role of the patient-practitioner relationship. Journal of rehabilitation medicine. 2014///. 46:153 |  |  | Y |  |  |  |
| J. Adsett,A. Hickey,A. Nagle,A.M. Mudge. Community-based maintenance exercise training following cardiac, pulmonary and heart failure rehabilitation: Feasibility, attendance predictors and scalability. European Journal of Heart Failure. 2014///. 16:63 |  | Y |  |  |  |  |
| Parminder K. Flora,Lawrence R. Brawley. Managing lapses in cardiac rehabilitation exercise therapy: examination of the problem-solving process. Rehabilitation psychology. 2013///. 58:369 |  | Y |  |  |  |  |
| Sussie Laustsen,Vibeke Elisabeth Hjortdal,Annemette Krintel Petersen. Predictors for not completing exercise-based rehabilitation following cardiac surgery. Scandinavian cardiovascular journal : SCJ. 2013///. 47:344 |  |  |  |  | Y |  |
| Su Yin Yang,Keng He Kong. Level and predictors of participation in patients with stroke undergoing inpatient rehabilitation. Singapore medical journal. 2013///. 54:564 |  |  |  |  |  |  |
| Mary Forhan,Brandon M. Zagorski,Susan Marzonlini,Paul Oh,David A. Alter. Predicting exercise adherence for patients with obesity and diabetes referred to a cardiac rehabilitation and secondary prevention program. Canadian journal of diabetes. 2013///. 37:189 |  |  | Y |  |  |  |
| Wendy M. Rodgers,Terra C. Murray,Anne Marie Selzler,Paul Norman. Development and impact of exercise self-efficacy types during and after cardiac rehabilitation. Rehabilitation psychology. 2013///. 58:178 |  |  | Y |  |  |  |
| Wan Ling Lee,Khatijah Lim Abdullah,Awang Mahmud Bulgiba,Imran Zainal Abidin. Prevalence and predictors of patient adherence to health recommendations after acute coronary syndrome: data for targeted interventions?. European journal of cardiovascular nursing : journal of the Working Group on Cardiovascular Nursing of the European Society of Cardiology. 2013///. 12:512 |  |  | Y |  |  |  |
| Conal Hayton,Allan Clark,Sandra Olive,Paula Browne,Penny Galey,Emma Knights,Lindi Staunton,Andrew Jones,Emma Coombes,Andrew M. Wilson. Barriers to pulmonary rehabilitation: characteristics that predict patient attendance and adherence. Respiratory medicine. 2013///. 107:401 |  |  |  |  | Y |  |
| Raffaele Griffo,Marco Ambrosetti,Roberto Tramarin,Francesco Fattirolli,Pier Luigi Temporelli,Anna Rita Vestri,Stefania De Feo,Luigi Tavazzi,investigators ICAROS. Effective secondary prevention through cardiac rehabilitation after coronary revascularization and predictors of poor adherence to lifestyle modification and medication. Results of the ICAROS Survey. International Journal of Cardiology. 2013///. 167:1390 |  | Y |  |  |  |  |
| Lisa Cadmus-Bertram,Alyson J. Littman,Cornelia M. Ulrich,Rachael Stovall,Rachel M. Ceballos,Bonnie A. McGregor,Ching Yun Wang,Jaya Ramaprasad,Anne McTiernan. Predictors of adherence to a 26-week viniyoga intervention among post-treatment breast cancer survivors. Journal of alternative and complementary medicine (New York, N.Y.). 2013///. 19:751 |  |  | Y |  |  |  |
| M.L. Garmendia,A.D. Dangour,C. Albala,P. Eguiguren,E. Allen,R. Uauy. Adherence to a physical activity intervention among older adults in a post-transitional middle income country: A quantitative and qualitative analysis. Journal of Nutrition, Health and Aging. 2013///. 17:466 |  |  |  | Y |  |  |
| S. Banerjee,K. Manley,L. Thomas,B. Shaw,J. Saxton,R. Mills,M. Rochester. Preoperative exercise protocol to aid recovery of radical cystectomy: Results of a feasibility study. European Urology, Supplements. 2013///. 12:125 |  |  |  |  |  | Abstract only |
| N.E. Allen,J. Song,C. Sherrington,S.R. Lord,V.S.C. Fung,J.C.T. Close,S.S. Paul,S.D. O'Rourke,S.M. Murray,C.G. Canning. Predictors of adherence to an exercise program in people with Parkinson's disease. Movement Disorders. 2013///. 28:S157 |  |  |  |  |  | Abstract only |
| Palmieri R. Macarone,P. Amodio,A. Feroce,M. Piciollo,R. Faggiani,R. Urgesi,C. Zampaletta. Effectiveness of fast-track rehabilitation in colorectal cancer resection for elderly patients: An italian single center preliminary experience. Digestive and Liver Disease. 2013///. 45:S187 |  | Y |  |  |  |  |
| Terra Murray,Wendy Rodgers. The Role of Socioeconomic Status and Control Beliefs on Frequency of Exercise During and After Cardiac Rehabilitation. Applied psychology.Health and well-being. 2012///. 4:49 |  |  | Y |  |  |  |
| Alexander M. Clark,Kathryn M. King-Shier,David R. Thompson,Melisa A. Spaling,Amanda S. Duncan,James A. Stone,Susan B. Jaglal,Jan E. Angus. A qualitative systematic review of influences on attendance at cardiac rehabilitation programs after referral. American heart journal. 2012///. 164:835 |  |  |  |  |  | Review |
| U. Muller-Buhl,P. Engeser,R. Leutgeb,J. Szecsenyi. Eligibility of patients with peripheral arterial disease for participation in a community-based walking exercise program: a single-centre experience. International angiology : a journal of the International Union of Angiology. 2012///. 31:462 |  | Y |  |  |  |  |
| Trine Moholdt,Mona Bekken Vold,Jostein Grimsmo,Stig Arild Slordahl,Ulrik Wisloff. Home-based aerobic interval training improves peak oxygen uptake equal to residential cardiac rehabilitation: a randomized, controlled trial. PloS one. 2012///. 7:e41199 |  |  | Y |  |  |  |
| Ru Yu Lien,Jeng Wei,Jiun Yi Li,Heng Hsin Tung,Chiang Yi Chen. [Difference in predictors of self efficacy and compliance between diabetic and non diabetic patients who underwent coronary artery bypass surgery]. Hu li za zhi The journal of nursing. 2012///. 59:40 |  |  |  |  |  | Chinese |
| Marijn de Bruin,Paschal Sheeran,Gerjo Kok,Anneke Hiemstra,Jan M. Prins,Harm J. Hospers,Gerard J.P. van Breukelen. Self-regulatory processes mediate the intention-behavior relation for adherence and exercise behaviors. Health psychology : official journal of the Division of Health Psychology, American Psychological Association. 2012///. 31:695 | Y |  |  |  |  |  |
| Jingjing Shang,Jennifer Wenzel,Sharon Krumm,Kathleen Griffith,Kerry Stewart. Who will drop out and who will drop in: exercise adherence in a randomized clinical trial among patients receiving active cancer treatment. Cancer nursing. 2012///. 35:312 |  |  | Y |  |  |  |
| Kerry S. Courneya,Kristina H. Karvinen,Margaret L. McNeely,Kristin L. Campbell,Sony Brar,Christy G. Woolcott,Anne McTiernan,Rachel Ballard-Barbash,Christine M. Friedenreich. Predictors of adherence to supervised and unsupervised exercise in the Alberta Physical Activity and Breast Cancer Prevention Trial. Journal of physical activity & health. 2012///. 9:857 |  |  |  | Y |  |  |
| M. Di Monaco,F. Vallero,E. De Toma,C. Castiglioni,L. Gardin,S. Giordano,R. Tappero. Adherence to recommendations for fall prevention significantly affects the risk of falling after hip fracture: post-hoc analyses of a quasi-randomized controlled trial. European journal of physical and rehabilitation medicine. 2012///. 48:9 |  | Y |  |  |  |  |
| Kerry S. Courneya,Clare Stevinson,Margaret L. McNeely,Christopher M. Sellar,Christine M. Friedenreich,Carolyn J. Peddle-McIntyre,Neil Chua,Tony Reiman. Predictors of follow-up exercise behavior 6 months after a randomized trial of supervised exercise training in lymphoma patients. Psycho-oncology. 2012///. 21:1124 |  |  | Y |  |  |  |
| Margaret L. McNeely,Matthew B. Parliament,Hadi Seikaly,Naresh Jha,David J. Magee,Mark J. Haykowsky,Kerry S. Courneya. Predictors of adherence to an exercise program for shoulder pain and dysfunction in head and neck cancer survivors. Supportive care in cancer : official journal of the Multinational Association of Supportive Care in Cancer. 2012///. 20:515 |  |  | Y |  |  |  |
| N.A. Azad,K. Bouchard,A. Mayhew,M. Carter,F.J. Molnar. Safety and predictors of adherence of a new rehabilitation program for older women with congestive heart failure. Journal of Geriatric Cardiology. 2012///. 9:243 |  |  |  |  |  | No multivariable |
| R. Wardini,A.K. Rizk,E. Chan-Thim,G. Moullec,Lorimier M. De,V. Pepin. Compliance to different exercise-training protocols in individuals with chronic obstructive pulmonary disease. American Journal of Respiratory and Critical Care Medicine. 2012///. 185:#pages# |  |  |  |  |  | Abstract only |
| Nancy E. Mayo,Liane Feldman,Susan Scott,Gerald Zavorsky,Do Jun Kim,Patrick Charlebois,Barry Stein,Francesco Carli. Impact of preoperative change in physical function on postoperative recovery: argument supporting prehabilitation for colorectal surgery. Surgery. 2011///. 150:505 |  | Y |  |  |  |  |
| Walter Swardfager,Nathan Herrmann,Susan Marzolini,Paul I. Oh,Mahwesh Saleem,Prathiba Shammi,Alexander Kiss,Jaclyn Cappell,Krista L. Lanctot. Verbal memory performance and completion of cardiac rehabilitation in patients with coronary artery disease. Psychosomatic medicine. 2011///. 73:580 |  |  | Y |  |  |  |
| Anne Tiedemann,Catherine Sherrington,Stephen R. Lord. Predictors of exercise adherence in older people living in retirement villages. Preventive medicine. 2011///. 52:480 |  |  |  |  |  | Letter to editor |
| Robert Wagenmakers,Martin Stevens,Johan W. Groothoff,Wiebren Zijlstra,Sjoerd K. Bulstra,Jan van Beveren,Jos J.A.M. van Raaij,Inge van den Akker-Scheek. Physical activity behavior of patients 1 year after primary total hip arthroplasty: a prospective multicenter cohort study. Physical therapy. 2011///. 91:373 | Y |  |  |  |  |  |
| Walter Swardfager,Nathan Herrmann,Susan Marzolini,Mahwesh Saleem,Shale B. Farber,Alexander Kiss,Paul I. Oh,Krista L. Lanctot. Major depressive disorder predicts completion, adherence, and outcomes in cardiac rehabilitation: a prospective cohort study of 195 patients with coronary artery disease. The Journal of clinical psychiatry. 2011///. 72:1181 |  |  | Y |  |  |  |
| Rita McGuire,Nancy Waltman,Lani Zimmerman. Intervention components promoting adherence to strength training exercise in breast cancer survivors with bone loss. Western journal of nursing research. 2011///. 33:671 |  |  | Y |  |  |  |
| Pauline T. Truong,Catherine A. Gaul,Rachel E. McDonald,Ross B. Petersen,Stuart O. Jones,Abraham S. Alexander,Jan T.W. Lim,Charles Ludgate. Prospective evaluation of a 12-week walking exercise program and its effect on fatigue in prostate cancer patients undergoing radical external beam radiotherapy. American journal of clinical oncology. 2011///. 34:350 |  | Y |  |  |  |  |
| M. Ta,J. George. Management of chronic obstructive pulmonary disease in Australia after the publication of national guidelines. Internal medicine journal. 2011///. 41:263 | Y |  |  |  |  |  |
| M. Bonnefoy,F. Boutitie,C. Mercier,F. Gueyffier,C. Carre,G. Guetemme,B. Ravis,M. Laville,C. Cornu. Efficacy of a home-based intervention programme on the physical activity level and functional ability of older people using domestic services: A randomised study. Journal of Nutrition, Health and Aging. 2011///. #volume#:1 |  |  |  | Y |  |  |
| Brooke Aggarwal,Ming Liao,Lori Mosca. Predictors of physical activity at 1 year in a randomized controlled trial of family members of patients with cardiovascular disease. The Journal of cardiovascular nursing. 2010///. 25:444 |  |  |  | Y |  |  |
| Lynn S. Kakos,Ashley J. Szabo,John Gunstad,Kelly M. Stanek,Donna Waechter,Joel Hughes,Faith Luyster,Richard Josephson,Jim Rosneck. Reduced executive functioning is associated with poorer outcome in cardiac rehabilitation. Preventive cardiology. 2010///. 13:100 |  | Y |  |  |  |  |
| Susan Marzolini,Henry Candelaria,Paul Oh. Prevalence and impact of musculoskeletal comorbidities in cardiac rehabilitation. Journal of Cardiopulmonary Rehabilitation and Prevention. 2010///. 30:391 |  |  | Y |  |  |  |
| Theresa M. Beckie,Jason W. Beckstead. Predicting cardiac rehabilitation attendance in a gender-tailored randomized clinical trial. Journal of Cardiopulmonary Rehabilitation and Prevention. 2010///. 30:147 |  |  | Y |  |  |  |
| Falko F. Sniehotta,Charlotta Gorski,Vera Araujo-Soares. Adoption of community-based cardiac rehabilitation programs and physical activity following phase III cardiac rehabilitation in Scotland: a prospective and predictive study. Psychology & health. 2010///. 25:839 |  |  | Y |  |  |  |
| Elizabeth R. Skidmore,Ellen M. Whyte,Margo B. Holm,James T. Becker,Meryl A. Butters,Mary Amanda Dew,Michael C. Munin,Eric J. Lenze. Cognitive and affective predictors of rehabilitation participation after stroke. Archives of physical medicine and rehabilitation. 2010///. 91:203 |  |  |  |  | Y |  |
| John Sharp,Claire Freeman. Patterns and predictors of uptake and adherence to cardiac rehabilitation. Journal of Cardiopulmonary Rehabilitation and Prevention. 2009///. 29:241 |  |  | Y |  |  |  |
| Rebecca N. Latka,Marty Alvarez-Reeves,Lisa Cadmus,Melinda L. Irwin. Adherence to a randomized controlled trial of aerobic exercise in breast cancer survivors: the Yale exercise and survivorship study. Journal of cancer survivorship : research and practice. 2009///. 3:148 |  |  | Y |  |  |  |
| Natalie A. Johnson,Lynette L.-Y. Lim,Steven J. Bowe. Multicenter randomized controlled trial of a home walking intervention after outpatient cardiac rehabilitation on health-related quality of life in women. European journal of cardiovascular prevention and rehabilitation : official journal of the European Society of Cardiology, Working Groups on Epidemiology & Prevention and Cardiac Rehabilitation and Exercise Physiology. 2009///. 16:633 |  |  | Y |  |  |  |
| Emily A. Kuhl,James A. Fauerbach,David E. Bush,Roy C. Ziegelstein. Relation of anxiety and adherence to risk-reducing recommendations following myocardial infarction. The American journal of cardiology. 2009///. 103:1629 | Y |  |  |  |  |  |
| Shu Yueh Chen,Ya Ling Tzeng. Path analysis for adherence to pelvic floor muscle exercise among women with urinary incontinence. The journal of nursing research : JNR. 2009///. 17:83 |  |  |  | Y |  |  |
| Kelly L. Russell,Steven R. Bray. Self-determined motivation predicts independent, home-based exercise following cardiac rehabilitation. Rehabilitation psychology. 2009///. 54:150 | Y | Y |  |  |  |  |
| Catherine Sherrington,Stephen R. Lord,Constance M. Vogler,Jacqueline C.T. Close,Kirsten Howard,Catherine M. Dean,Lindy Clemson,Elizabeth Barraclough,Elisabeth Ramsay,Sandra D. O'Rourke,Robert G. Cumming. Minimising disability and falls in older people through a post-hospital exercise program: a protocol for a randomised controlled trial and economic evaluation. BMC geriatrics. 2009///. 9:8 |  |  |  |  |  | Protocol |
| A. Soleimani,A. Abbasi,M. Nejatian,M. Salarifar,S. Darabian,A.A. Karimi,S. Davoodi,S.E. Kassaian,S.H. Abbasi,M. Sheikhfathollahi. Factors predicting discontinuation of a hospital-based cardiac rehabilitation programme. Kardiologia Polska. 2009///. 67:140 |  |  | Y |  |  |  |
| S.M. Dunlay,B.J. Witt,T.G. Allison,S.N. Hayes,S.A. Weston,E. Koepsell,V.L. Roger. Barriers to participation in cardiac rehabilitation. American heart journal. 2009///. 158:852 |  |  | Y |  |  |  |
| M. Invernizzi,M. Massara,G.S. Viscontini,M. Bellotti,C. Ricupero,S. Carda,C. Cisari. Osteoporotic hip fracture in the fragile elderly, rehabilitation and autonomy: The PerFem study. Bone. 2009///. 44:S411 |  |  |  |  |  | Abstract only |
| Gerard J. Molloy,Linda Perkins-Porras,Mimi R. Bhattacharyya,Philip C. Strike,Andrew Steptoe. Practical support predicts medication adherence and attendance at cardiac rehabilitation following acute coronary syndrome. Journal of Psychosomatic Research. 2008///. 65:581 |  |  | Y |  |  |  |
| Susan Marzolini,Dina Brooks,Paul I. Oh. Sex differences in completion of a 12-month cardiac rehabilitation programme: an analysis of 5922 women and men. European journal of cardiovascular prevention and rehabilitation : official journal of the European Society of Cardiology, Working Groups on Epidemiology & Prevention and Cardiac Rehabilitation and Exercise Physiology. 2008///. 15:698 |  |  | Y |  |  |  |
| Richard L. Skolasky,Ellen J. Mackenzie,Stephen T. Wegener,Lee H. Riley. Patient activation and adherence to physical therapy in persons undergoing spine surgery. Spine. 2008///. 33:E784 |  |  | Y | Y |  |  |
| Kirsten M. Nielsen,Ole Faergeman,Anders Foldspang,Mogens L. Larsen. Cardiac rehabilitation: health characteristics and socio-economic status among those who do not attend. European journal of public health. 2008///. 18:479 |  |  | Y |  |  |  |
| Kerry S. Courneya,Roanne J. Segal,Karen Gelmon,Robert D. Reid,John R. Mackey,Christine M. Friedenreich,Caroline Proulx,Kirstin Lane,Aliya B. Ladha,Jeffrey K. Vallance,Donald C. McKenzie. Predictors of supervised exercise adherence during breast cancer chemotherapy. Medicine and science in sports and exercise. 2008///. 40:1180 |  |  | Y |  |  |  |
| Kerry S. Courneya,Donald C. McKenzie,Robert D. Reid,John R. Mackey,Karen Gelmon,Christine M. Friedenreich,Aliya B. Ladha,Caroline Proulx,Kirstin Lane,Jeffrey K. Vallance,Roanne J. Segal. Barriers to supervised exercise training in a randomized controlled trial of breast cancer patients receiving chemotherapy. Annals of behavioral medicine : a publication of the Society of Behavioral Medicine. 2008///. 35:116 |  |  | Y |  |  |  |
| Ramsey Sabit,Timothy L. Griffiths,Alan J. Watkins,Wendy Evans,Charlotte E. Bolton,Dennis J. Shale,Keir E. Lewis. Predictors of poor attendance at an outpatient pulmonary rehabilitation programme. Respiratory medicine. 2008///. 102:819 |  |  |  |  | Y |  |
| Gerard J. Molloy,Linda Perkins-Porras,Philip C. Strike,Andrew Steptoe. Social networks and partner stress as predictors of adherence to medication, rehabilitation attendance, and quality of life following acute coronary syndrome. Health psychology : official journal of the Division of Health Psychology, American Psychological Association. 2008///. 27:52 |  |  | Y |  |  |  |
| R. Schwarzer,A. Luszczynska,J.P. Ziegelmann,U. Scholz,S. Lippke. Social-Cognitive Predictors of Physical Exercise Adherence: Three Longitudinal Studies in Rehabilitation. Health Psychology. 2008///. 27:S54 |  |  |  |  |  | Review |
| Barbara Resnick,Denise Orwig,Christopher D'Adamo,Janet Yu-Yahiro,William Hawkes,Michelle Shardell,Justine Golden,Sheryl Zimmerman,Jay Magaziner. Factors that influence exercise activity among women post hip fracture participating in the Exercise Plus Program. Clinical interventions in aging. 2007///. 2:413 |  | Y |  |  |  |  |
| Mark D. Bishop,John Meuleman,Michael Robinson,Kathye E. Light. Influence of pain and depression on fear of falling, mobility, and balance in older male veterans. Journal of rehabilitation research and development. 2007///. 44:675 |  |  |  |  | Y |  |
| DorAnne Donesky-Cuenco,Susan Janson,John Neuhaus,Torsten B. Neilands,Virginia Carrieri-Kohlman. Adherence to a home-walking prescription in patients with chronic obstructive pulmonary disease. Heart & lung : the journal of critical care. 2007///. 36:348 |  | Y |  |  |  |  |
| Alyson B. Moadel,Chirag Shah,Judith Wylie-Rosett,Melanie S. Harris,Sapana R. Patel,Charles B. Hall,Joseph A. Sparano. Randomized controlled trial of yoga among a multiethnic sample of breast cancer patients: effects on quality of life. Journal of clinical oncology : official journal of the American Society of Clinical Oncology. 2007///. 25:4387 |  |  | Y |  |  |  |
| J.P. Ziegelmann,S. Lippke. Planning and strategy use in health behavior change: A life span view. International journal of behavioral medicine. 2007///. 14:30 |  |  |  | Y |  |  |
| L. Yardley,M. Donovan-Hall. Predicting adherence to exercise-based therapy in rehabilitation. Rehabilitation psychology. 2007///. 52:56 |  |  |  | Y |  |  |
| Ian M. Kronish,Nina Rieckmann,Ethan A. Halm,Daichi Shimbo,David Vorchheimer,Donald C. Haas,Karina W. Davidson. Persistent depression affects adherence to secondary prevention behaviors after acute coronary syndromes. Journal of General Internal Medicine. 2006///. 21:1178 |  |  | Y |  |  |  |
| Rebecca Forkan,Breeanna Pumper,Nicole Smyth,Hilary Wirkkala,Marcia A. Ciol,Anne Shumway-Cook. Exercise adherence following physical therapy intervention in older adults with impaired balance. Physical therapy. 2006///. 86:401 |  | Y |  |  | Y |  |
| Neil Smart,Brian Haluska,Leanne Jeffriess,Thomas H. Marwick. Predictors of a sustained response to exercise training in patients with chronic heart failure: a telemonitoring study. American heart journal. 2005///. 150:1240 |  |  | Y |  |  |  |
| David P. French,Robert J.P. Lewin,Nina Watson,David R. Thompson. Do illness perceptions predict attendance at cardiac rehabilitation and quality of life following myocardial infarction?. Journal of Psychosomatic Research. 2005///. 59:315 |  |  | Y |  |  |  |
| Sheri L. Maddigan,Sumit R. Majumdar,Jeffrey A. Johnson. Understanding the complex associations between patient-provider relationships, self-care behaviours, and health-related quality of life in type 2 diabetes: a structural equation modeling approach. Quality of life research : an international journal of quality of life aspects of treatment, care and rehabilitation. 2005///. 14:1489 |  |  | Y |  |  |  |
| Kathleen K. Mangione,Rebecca L. Craik,Susan S. Tomlinson,Kerstin M. Palombaro. Can elderly patients who have had a hip fracture perform moderate- to high-intensity exercise at home?. Physical therapy. 2005///. 85:727 |  |  |  | Y |  |  |
| Molly Byrne,Jane Walsh,Andrew W. Murphy. Secondary prevention of coronary heart disease: patient beliefs and health-related behaviour. Journal of Psychosomatic Research. 2005///. 58:403 | Y | Y |  |  |  |  |
| Karen Harkness,Kelly M. Smith,Lisa Taraba,Cheri L. Mackenzie,Elizabeth Gunn,Heather M. Arthur. Effect of a postoperative telephone intervention on attendance at intake for cardiac rehabilitation after coronary artery bypass graft surgery. Heart & lung : the journal of critical care. 2005///. 34:179 |  |  | Y |  |  |  |
| Anthony Lyngcoln,Nicholas Taylor,Tania Pizzari,Kris Baskus. The relationship between adherence to hand therapy and short-term outcome after distal radius fracture. Journal of hand therapy : official journal of the American Society of Hand Therapists. 2005///. 18:2 |  |  |  | Y |  |  |
| S Nicole Culos-Reed,Christopher Shields,Lawrence R. Brawley. Breast cancer survivors involved in vigorous team physical activity: psychosocial correlates of maintenance participation. Psycho-oncology. 2005///. 14:594 |  |  | Y |  |  |  |
| M. Milne,C. Hall,L. Forwell. Self-efficacy, imagery use, and adherence to rehabilitation by injured athletes. Journal of Sport Rehabilitation. 2005///. 14:150 |  |  | Y |  |  |  |
| A.M. Jette,J. Keysor,W. Coster,P. Ni,S. Haley. Beyond function: Predicting participation in a rehabilitation cohort. Archives of physical medicine and rehabilitation. 2005///. 86:2087 |  |  |  | Y |  |  |
| S. Jennings,D. Carey. Capacity and equity in cardiac rehabilitation in the eastern region: good and bad news. Irish journal of medical science. 2004///. 173:151 |  |  | Y |  |  |  |
| Teresita Corvera-Tindel,Lynn V. Doering,Teresita Gomez,Kathleen Dracup. Predictors of noncompliance to exercise training in heart failure. The Journal of cardiovascular nursing. 2004///. 19:269 |  |  | Y |  |  |  |
| Andria M. Ratchford,Richard F. Hamman,Judith G. Regensteiner,David J. Magid,Stacy Brennan Gallagher,John A. Merenich. Attendance and graduation patterns in a group-model health maintenance organization alternative cardiac rehabilitation program. Journal of cardiopulmonary rehabilitation. 2004///. 24:150 |  |  |  |  | Y |  |
| Eric J. Lenze,Michael C. Munin,Mary Amanda Dew,Joan C. Rogers,Karen Seligman,Benoit H. Mulsant,Charles F. Reynolds. Adverse effects of depression and cognitive impairment on rehabilitation participation and recovery from hip fracture. International Journal of Geriatric Psychiatry. 2004///. 19:472 |  |  |  |  |  | No multivariable |
| Joshua Fogel,James A. Fauerbach,Roy C. Ziegelstein,David E. Bush. Quality of life in physical health domains predicts adherence among myocardial infarction patients even after adjusting for depressive symptoms. Journal of Psychosomatic Research. 2004///. 56:75 |  |  | Y |  |  |  |
| K.S. Courneya,C.M. Friedenreich,H.A. Quinney,A.L.A. Fields,L.W. Jones,A.S. Fairey. Predictors of adherence and contamination in a randomized trial of exercise in colorectal cancer survivors. Psycho-oncology. 2004///. 13:857 |  |  | Y |  |  |  |
| L. Husak,H.M. Krumholz,Z.Q. Lin,S.V. Kasl,J.A. Mattera,S.A. Roumanis,V. Vaccarino. Social Support as a Predictor of Participation in Cardiac Rehabilitation after Coronary Artery Bypass Graft Surgery. Journal of cardiopulmonary rehabilitation. 2004///. 24:19 |  |  | Y |  |  |  |
| V. Sundararajan,S.J. Bunker,S. Begg,R. Marshall,H. Burney. Attendance rates and outcomes of cardiac rehabilitation in Victoria, 1998. Medical Journal of Australia. 2004///. 180:268 |  | Y |  |  |  |  |
| Robyn L. Farley,Tracey D. Wade,Libby Birchmore. Factors influencing attendance at cardiac rehabilitation among coronary heart disease patients. European journal of cardiovascular nursing : journal of the Working Group on Cardiovascular Nursing of the European Society of Cardiology. 2003///. 2:205 |  |  | Y |  |  |  |
| Dianne Alewijnse,Ilse Mesters,Job Metsemakers,Bart van den Borne. Predictors of long-term adherence to pelvic floor muscle exercise therapy among women with urinary incontinence. Health education research. 2003///. 18:511 |  |  |  | Y |  |  |
| Marten Munneke,Zuzana de Jong,Aeilko H. Zwinderman,Annemarie Jansen,H.Karel Ronday,Wilfred F.H. Peter,Dies C.G. Boonman,Cornelia H.M. van den Ende,Theodora P.M. Vliet Vlieland,Johanna M.W. Hazes. Adherence and satisfaction of rheumatoid arthritis patients with a long-term intensive dynamic exercise program (RAPIT program). Arthritis and rheumatism. 2003///. 49:665 |  |  | Y |  |  |  |
| Anya Whitmarsh,Maria Koutantji,Kate Sidell. Illness perceptions, mood and coping in predicting attendance at cardiac rehabilitation. British journal of health psychology. 2003///. 8:209 |  |  | Y |  |  |  |
| Bernice C. Yates,Jana L. Braklow-Whitton,Sangeeta Agrawal. Outcomes of cardiac rehabilitation participants and nonparticipants in a rural area. Rehabilitation nursing : the official journal of the Association of Rehabilitation Nurses. 2003///. 28:57 |  |  |  |  | Y |  |
| Shirley M. Moore,Mary A. Dolansky,Cornelia M. Ruland,Fredric J. Pashkow,Gordon G. Blackburn. Predictors of women's exercise maintenance after cardiac rehabilitation. Journal of cardiopulmonary rehabilitation. 2003///. 23:40 |  |  | Y |  |  |  |
| Kerry S. Courneya,Christine M. Friedenreich,Rami A. Sela,H.Arthur Quinney,Ryan E. Rhodes. Correlates of adherence and contamination in a randomized controlled trial of exercise in cancer survivors: an application of the theory of planned behavior and the five factor model of personality. Annals of behavioral medicine : a publication of the Society of Behavioral Medicine. 2002///. 24:257 |  |  | Y |  |  |  |
| A.F. Cooper,G. Jackson,J. Weinman,R. Horne. Factors associated with cardiac rehabilitation attendance: a systematic review of the literature. Clinical rehabilitation. 2002///. 16:541 |  |  |  |  |  | Review |
| J.J. Carlson,G.J. Norman,D.L. Feltz,B.A. Franklin,J.A. Johnson,S.K. Locke. Self-efficacy, psychosocial factors, and exercise behavior in traditional versus modified cardiac rehabilitation. Journal of cardiopulmonary rehabilitation. 2001///. 21:363 |  |  | Y |  |  |  |
| D. Lane,D. Carroll,C. Ring,D.G. Beevers,G.Y. Lip. Predictors of attendance at cardiac rehabilitation after myocardial infarction. Journal of Psychosomatic Research. 2001///. 51:497 |  |  | Y |  |  |  |
| W.G. Hundley,D.W. Kitzman,T.M. Morgan,C.A. Hamilton,S.N. Darty,K.P. Stewart,D.M. Herrington,K.M. Link,W.C. Little. Cardiac cycle-dependent changes in aortic area and distensibility are reduced in older patients with isolated diastolic heart failure and correlate with exercise intolerance. Journal of the American College of Cardiology. 2001///. 38:796 |  | Y |  |  |  |  |
| L.S. Evangelista,J. Berg,K. Dracup. Relationship between psychosocial variables and compliance in patients with heart failure. Heart & lung : the journal of critical care. 2001///. 30:294 |  |  | Y |  |  |  |
| K.M. King,D.P. Humen,H.L. Smith,C.L. Phan,K.K. Teo. Predicting and explaining cardiac rehabilitation attendance. The Canadian journal of cardiology. 2001///. 17:291 |  |  |  |  |  | Age unclear |
| E. Missik. Women and cardiac rehabilitation: accessibility issues and policy recommendations. Rehabilitation nursing : the official journal of the Association of Rehabilitation Nurses. 2001///. 26:141 |  |  |  |  |  | Non participation |
| C.-P. Lai,C. Lee,J.-H. Wang,J.-J. Hsieh,C.-M. Lin. Comparison of time-related changes between body weight and subsequent effects of supervised cardiac rehabilitation in patients with acute myocardial infarction. Tzu Chi Medical Journal. 2001///. 13:203 |  |  |  |  |  | Chinese |
| C.Y. Chen,P.S. Neufeld,C.A. Feely,C.S. Skinner. Factors influencing compliance with home exercise programs among patients with upper-extremity impairment. The American journal of occupational therapy : official publication of the American Occupational Therapy Association. 1999///. 53:171 |  |  |  | Y |  |  |
| A. Cooper,G. Lloyd,J. Weinman,G. Jackson. Why patients do not attend cardiac rehabilitation: Role of intentions and illness beliefs. Heart (British Cardiac Society). 1999///. 82:234 |  |  | Y |  |  |  |
| D.K. Taylor,K.R. Barber,B.A. Mcintosh,M. Khan. The impact of post acute myocardial infarction (AMI) depression on patient compliance and risk factor modification. Psychology, Health and Medicine. 1998///. 3:439 |  |  | Y |  |  |  |
| N.A. Johnson,R.F. Heller. Prediction of patient nonadherence with home-based exercise for cardiac rehabilitation: The role of perceived barriers and perceived benefits. Preventive medicine. 1998///. 27:56 | Y |  |  |  |  |  |
| K.E. Bendstrup,J. Ingemann Jensen,S. Holm,B. Bengtsson. Out-patient rehabilitation improves activities of daily living, quality of life and exercise tolerance in chronic obstructive pulmonary disease. The European respiratory journal. 1997///. 10:2801 |  | Y | Y |  |  |  |
| W.J. Rejeski,L.R. Brawley,W. Ettinger,T. Morgan,C. Thompson. Compliance to exercise therapy in older participants with knee osteoarthritis: implications for treating disability. Medicine and science in sports and exercise. 1997///. 29:977 |  |  |  | Y |  |  |
| E.A. Hellman. Use of the stages of change in exercise adherence model among older adults with a cardiac diagnosis. Journal of cardiopulmonary rehabilitation. 1997///. 17:145 | Y |  |  |  |  |  |
| C.H. Stenstrom,B. Arge,A. Sundbom. Home exercise and compliance in inflammatory rheumatic diseases--a prospective clinical trial. The Journal of rheumatology. 1997///. 24:470 |  |  | Y |  |  |  |
| C. Jeng,L.T. Braun. The influence of self-efficacy on exercise intensity, compliance rate and cardiac rehabilitation outcomes among coronary artery disease patients. Progress in cardiovascular nursing. 1997///. 12:13 |  | Y |  |  |  |  |
| J. Roomi,M.M. Johnson,K. Waters,A. Yohannes,A. Helm,M.J. Connolly. Respiratory rehabilitation, exercise capacity and quality of life in chronic airways disease in old age. Age and ageing. 1996///. 25:12 |  | Y |  |  |  |  |
| J. van Dixhoorn,H.J. Duivenvoorden,J. Pool. Success and failure of exercise training after myocardial infarction: is the outcome predictable?. Journal of the American College of Cardiology. 1990///. 15:974 |  |  | Y |  |  |  |
| P. Miller,R. Wikoff,M. McMahon,M.J. Garrett,K. Ringel. Influence of a nursing intervention on regimen adherence and societal adjustments postmyocardial infarction. Nursing research. 1988///. 37:297 |  | Y |  |  |  |  |
| R.M. Kaplan,S.L. Hartwell. Differential effects of social support and social network on physiological and social outcomes in men and women with type II diabetes mellitus. Health psychology : official journal of the Division of Health Psychology, American Psychological Association. 1987///. 6:387 |  |  | Y |  |  |  |
| W. Wilson,D.V. Ary,A. Biglan,R.E. Glasgow,D.J. Toobert,D.R. Campbell. Psychosocial predictors of self-care behaviors (compliance) and glycemic control in non-insulin-dependent diabetes mellitus. Diabetes care. 1986///. 9:614 | Y |  |  |  |  |  |
| A.F. Fontana,R.D. Kerns,R.L. Rosenberg. Exercise training for cardiac patients: Adherence, fitness, and benefits. Journal of cardiopulmonary rehabilitation. 1986///. 6:4 |  |  | Y |  |  |  |
| Andria R. Morielli, Normand G. Boulé, Nawaid Usmani, Kurian Joseph, Keith Tankel, Diane Severin, Kerry S. Courneya. Predictors of adherence to aerobic exercise in rectal cancer patients during and after neoadjuvant chemoradiotherapy. Psychology, Health & Medicine. 2018. 23:224-231 |  |  | Y |  |  | No multivariable |
| E. Hile, R. Neuhold, V. Davidson. TRAINING FOR THE FIGHT: ADHERENCE TO A NOVEL PREHAB APPROACH IN PANCREATICODUODENECTOMY. Rehabilitation Oncology. 2018. 36:E8-E8 |  |  |  |  |  | Abstract only |
| Sebastian Weeger, Erik Farin. The effect of the patient–physician relationship on health-related quality of life after cardiac rehabilitation. Disability & Rehabilitation. 2017. 39:468-476 |  |  | Y |  |  |  |
| A. Soto-Varela, A. Faraldo-García, M. Del-Río-Valeiras, M. Rossi-Izquierdo, I. Vaamonde-Sánchez-Andrade, P. Gayoso-Diz, A. Lirola-Delgado, S. Santos-Pérez. Adherence of older people with instability in vestibular rehabilitation programmes: prediction criteria. Journal of Laryngology & Otology. 2017. 131:232-238 |  |  |  |  |  | No multivariable |
| Jon Arne Sandmæl, Asta Bye, Tora Skeidsvoll Solheim, Guro Birgitte Stene, Lene Thorsen, Stein Kaasa, Jo-Åsmund Lund, Line Merethe Oldervoll, Jon Arne Sandmael, Jo-Åsmund Lund. Feasibility and preliminary effects of resistance training and nutritional supplements during versus after radiotherapy in patients with head and neck cancer: A pilot randomized trial. Cancer (0008543X). 2017. 123:4440-4448 |  |  | Y |  |  |  |
| Laura Rogers, Kerry Courneya, Phillip Anton, Patricia Hopkins-Price, Steven Verhulst, Randall Robbs, Sandra Vicari, Edward McAuley, Laura Q. Rogers, Kerry S. Courneya, Phillip M. Anton, Randall S. Robbs, Sandra K. Vicari. Social Cognitive Constructs Did Not Mediate the BEAT Cancer Intervention Effects on Objective Physical Activity Behavior Based on Multivariable Path Analysis. Annals of Behavioral Medicine. 2017. 51:321-326 |  |  | Y |  |  |  |
| Noah Ivers, J. D. Schwalm, Holly O. Witteman, Justin Presseau, Monica Taljaard, Tara McCready, Beth Bosiak, Jennifer Cunningham, Shelley Smarz, Laura Desveaux, Jack V. Tu, Clare Atzema, Garth Oakes, Wanrudee Isaranuwatchai, Sherry L. Grace, R. Sacha Bhatia, Madhu Natarajan, Jeremy M. Grimshaw. Interventions Supporting Long-term Adherence aNd Decreasing cardiovascular events (ISLAND): Pragmatic randomized trial protocol. American Heart Journal. 2017. 190:64-75 |  |  |  |  |  | Protocol |
| Anne Kirstine Eriksen, Rikke Dalgaard Hansen, Michael Borre, Ryan Godsk Larsen, Jeppe Munthe Jensen, Kristian Overgaard, Mette Borre, Cecilie Kyrø, Rikard Landberg, Anja Olsen, Anne Tjønneland. A lifestyle intervention among elderly men on active surveillance for non-aggressive prostate cancer: a randomised feasibility study with whole-grain rye and exercise. Trials. 2017. 18:1-12 |  |  | Y |  |  |  |
| Abebaw Mengistu Yohannes, Patrick J. Raue, Dora Kanellopoulos, Amanda McGovern, Jo Anne Sirey, Dimitris N. Kiosses, Samprit Banerjee, Joanna K. Seirup, Richard S. Novitch, George S. AleManchester, George S. Alexopoulos. Predictors of All-Cause Mortality in Patients With Severe COPD and Major Depression Admitted to a Rehabilitation Hospital. CHEST. 2016. 149:467-473 |  | Y |  |  |  |  |
| Y. A. Tuakli-Wosornu, F. Selzer, E. Losina, J. N. Katz. Predictors of Exercise Adherence in Patients With Meniscal Tear and Osteoarthritis. Archives of physical medicine and rehabilitation. 2016. 97:1945-1952 |  |  | Y |  |  |  |
| I. Svege, L. Fernandes, L. Nordsletten, I. Holm, M. A. Risberg. Long-Term Effect of Exercise Therapy and Patient Education on Impairments and Activity Limitations in People With Hip Osteoarthritis: secondary Outcome Analysis of a Randomized Clinical Trial. Physical therapy. 2016. 96:818-827 |  | Y |  |  |  |  |
| Daniela Stan, Katrina Croghan, Ivana Croghan, Sarah Jenkins, Stephanie Sutherland, Andrea Cheville, Sandhya Pruthi, Daniela L. Stan, Katrina A. Croghan, Ivana T. Croghan, Sarah M. Jenkins, Stephanie J. Sutherland, Andrea L. Cheville. Randomized pilot trial of yoga versus strengthening exercises in breast cancer survivors with cancer-related fatigue. Supportive Care in Cancer. 2016. 24:4005-4015 |  | Y |  |  |  |  |
| Marilyn L. Moy, Carlos H. Martinez, Reema Kadri, Pia Roman, Robert G. Holleman, Hyungjin Myra Kim, Huong Q. Nguyen, Miriam D. Cohen, David E. Goodrich, Nicholas D. Giardino, Caroline R. Richardson. Long-Term Effects of an Internet-Mediated Pedometer-Based Walking Program for Chronic Obstructive Pulmonary Disease: Randomized Controlled Trial. Journal of Medical Internet Research. 2016. 18:35-48 |  | Y |  |  |  |  |
| Alexander Mertens, Christopher Brandl, Talya Miron-Shatz, Christopher Schlick, Till Neumann, Andreas Kribben, Sven Meister, Clarissa Jonas Diamantidis, Urs-Vito Albrecht, Peter Horn, Stefan Becker. A mobile application improves therapy-adherence rates in elderly patients undergoing rehabilitation: A crossover design study comparing documentation via iPad with paper-based control. Medicine. 2016. 95:1-8 |  | Y |  |  |  |  |
| Andrew McLachlan, Fiona Doolan Noble, Mildred Lee, Katherine McLean, Andrew Kerr, Andy McLachlan, Fiona Doolan-Noble, Andrew J. Kerr. The electronic tracking of referral and attendance at cardiac rehabilitation in Counties Manukau Health: a potential model for New Zealand. New Zealand Medical Journal. 2016. 129:64-71 |  |  | Y |  |  |  |
| Jo Ann G. Kim. Phase II Cardiac Rehabilitation Participants' Perception of Nurse Caring Correlated With Participants' Depression, Anxiety, and Adherence. International Journal for Human Caring. 2016. 20:213-219 |  |  |  |  |  | Dissertation |
| K. J. Franke, U. Domanski, M. Schroeder, V. Jansen, F. Artmann, U. Weber, R. Ettler, G. Nilius. Telemonitoring of home exercise cycle training in patients with COPD. International journal of chronic obstructive pulmonary disease. 2016. 11:2821-2829 |  | Y |  |  |  |  |
| Lauren C. Capozzi, Margaret L. McNeely, Harold Y. Lau, Raylene A. Reimer, Janine Giese-Davis, Tak S. Fung, S. Nicole Culos-Reed. Patient-reported outcomes, body composition, and nutrition status in patients with head and neck cancer: Results from an exploratory randomized controlled exercise trial. Cancer (0008543X). 2016. 122:1185-1200 |  |  | Y |  |  |  |
| Maria Bäck, Åsa Cider, Johan Herlitz, Mari Lundberg, Bengt Jansson. Kinesiophobia mediates the influences on attendance at exercise-based cardiac rehabilitation in patients with coronary artery disease. Physiotherapy Theory & Practice. 2016. 32:571-580 |  |  |  |  |  |  |
| Laura Q. Rogers, Amanda Fogleman, Steven Verhulst, Randall Robbs, Mudita Bhugra, Krishna Rao, James Malone, K. Thomas Robbins. Refining Measurement of Social Cognitive Theory Factors Associated with Exercise Adherence in Head and Neck Cancer Patients. Journal of Psychosocial Oncology. 2015. 33:467-487 |  |  | Y |  |  |  |
| R. J. McNamara, Z. J. McKeough, D. K. McKenzie, J. A. Alison. Acceptability of the aquatic environment for exercise training by people with chronic obstructive pulmonary disease with physical comorbidities: additional results from a randomised controlled trial. Physiotherapy. 2015. 101:187-192 |  | Y |  |  |  |  |
| Monika E. Slovinec D'Angelo, Luc G. Pelletier, Robert D. Reid, Veronika Huta. The Roles of Self-Efficacy and Motivation in the Prediction of Short- and Long-Term Adherence to Exercise Among Patients With Coronary Heart Disease. Health Psychology. 2014. 33:1344-1353 |  |  | Y |  |  |  |
| A. McGrady, R. Burkes, D. Badenhop, R. McGinnis. Effects of a brief intervention on retention of patients in a cardiac rehabilitation program. Applied psychophysiology and biofeedback. 2014. 39:163-70 |  |  | Y |  |  |  |
| E. Madssen, I. Arbo, I. Granoien, L. Walderhaug, T. Moholdt. Peak oxygen uptake after cardiac rehabilitation: a randomized controlled trial of a 12-month maintenance program versus usual care. PloS one. 2014. 9:e107924 |  |  | Y |  |  |  |
| G. S. Alexopoulos, D. N. Kiosses, J. A. Sirey, D. Kanellopoulos, J. K. Seirup, R. S. Novitch, S. Ghosh, S. Banerjee, P. J. Raue. Untangling therapeutic ingredients of a personalized intervention for patients with depression and severe COPD. American journal of geriatric psychiatry. 2014. 22:1316-1324 |  | Y |  |  |  |  |
| S. Y. Yang, K. H. Kong, Su-Yin Yang, Keng He Kong. Level and predictors of participation in patients with stroke undergoing inpatient rehabilitation. Singapore Medical Journal. 2013. 54:564-568 |  |  | Y |  |  |  |
| Sarah Pakzad, Yannick Charette, Paul Bourque. Liens entre la perception de la maladie cardiovasculaire et la participation aux programmes de rÃ©adaptation cardiaque : une Ã©tude exploratoire. Canadian Journal of Cardiovascular Nursing. 2013. 23:19-25 |  |  | Y |  |  |  |
| Alison M. Mudge, Julie Adsett. Factors Predicting Successful Transition to Community-based Maintenance Exercise Programs Following Exercise Rehabilitation. Cardiopulmonary Physical Therapy Journal (American Physical Therapy Association, Cardiopulmonary Section). 2013. 24:18-24 |  |  |  |  | Y |  |
| Joanne McDonall, Mari Botti, Bernice Redley, Beverley Wood. Patient participation in a cardiac rehabilitation program. Journal of Cardiopulmonary Rehabilitation & Prevention. 2013. 33:185-188 |  |  |  |  | Y |  |
| L. Cadmus-Bertram, A. J. Littman, C. M. Ulrich, R. Stovall, R. M. Ceballos, B. A. McGregor, C. Y. Wang, J. Ramaprasad, A. McTiernan. Predictors of adherence to a 26-week viniyoga intervention among post-treatment breast cancer survivors. Journal of alternative and complementary medicine (new york, N.Y.). 2013. 19:751-758 |  |  | Y |  |  |  |
| R. Wardini, A. K. Rizk, E. Chan-Thim, G. Moullec, M. De Lorimier, V. Pepin. Compliance to different exercise-training protocols in individuals with chronic obstructive pulmonary disease. American journal of respiratory and critical care medicine. 2012. 185:#pages# |  |  |  |  |  | Dissertation |
| B. Steele, C. Dougherty, R. Burr, I. Gylys-Colwell, J. Hunziker. An intervention to enhance function in severe cardiopulmonary illness. American journal of respiratory and critical care medicine. 2012. 185:#pages# |  |  |  |  |  | Abstract only |
| J. Shang, J. Wenzel, S. Krumm, K. Griffith, K. Stewart. Who will drop out and who will drop in: exercise adherence in a randomized clinical trial among patients receiving active cancer treatment. Cancer nursing. 2012. 35:312-322 |  |  | Y |  |  |  |
| T. Moholdt, M. Bekken Vold, J. Grimsmo, S. A. Slordahl, U. Wisloff. Home-based aerobic interval training improves peak oxygen uptake equal to residential cardiac rehabilitation: a randomized, controlled trial. Plos one. 2012. 7:e41199 |  |  | Y |  |  |  |
| M. L. McNeely, M. B. Parliament, H. Seikaly, N. Jha, D. J. Magee, M. J. Haykowsky, K. S. Courneya, Margaret L. McNeely, Matthew B. Parliament, Hadi Seikaly, Naresh Jha, David J. Magee, Mark J. Haykowsky, Kerry S. Courneya. Predictors of adherence to an exercise program for shoulder pain and dysfunction in head and neck cancer survivors. Supportive Care in Cancer. 2012. 20:515-522 |  |  | Y |  |  |  |
| Gerald Choon-Huat Koh, Sanjiv K. Saxena, Tze-Pin Ng, David Yong, Ngan-Phoon Fong. Effect of Duration, Participation Rate, and Supervision During Community Rehabilitation on Functional Outcomes in the First Poststroke Year in Singapore. Archives of Physical Medicine & Rehabilitation. 2012. 93:279-286 |  | Y |  |  |  |  |
| K. S. Courneya, C. Stevinson, M. L. McNeely, C. M. Sellar, C. M. Friedenreich, C. J. Peddle-McIntyre, N. Chua, T. Reiman. Predictors of follow-up exercise behavior 6 months after a randomized trial of supervised exercise training in lymphoma patients. Psycho-oncology. 2012. 21:1124-1131 |  |  | Y |  |  |  |
| K. S. Courneya, K. H. Karvinen, M. L. McNeely, K. L. Campbell, S. Brar, C. G. Woolcott, A. McTiernan, R. Ballard-Barbash, C. M. Friedenreich. Predictors of adherence to supervised and unsupervised exercise in the Alberta Physical Activity and Breast Cancer Prevention Trial. Journal of physical activity & health. 2012. 9:857-66 |  |  | Y |  |  |  |
| W. Swardfager, N. Herrmann, S. Marzolini, P. I. Oh, M. Saleem, P. Shammi, A. Kiss, J. Cappell, K. L. Lanctôt. Verbal memory performance and completion of cardiac rehabilitation in patients with coronary artery disease. Psychosomatic Medicine. 2011. 73:580-587 |  |  | Y |  |  |  |
| S. Marzolini, H. Candelaria, P. Oh. Prevalence and impact of musculoskeletal comorbidities in cardiac rehabilitation. Journal of Cardiopulmonary Rehabilitation & Prevention. 2010. 30:391-400 |  |  | Y |  |  |  |
| N. A. Johnson, K. J. Inder, A. L. Nagle, J. H. Wiggers. Attendance at outpatient cardiac rehabilitation: is it enhanced by specialist nurse referral?. Australian Journal of Advanced Nursing. 2010. 27:31-37 |  |  | Y |  |  |  |
| P. Fitzpatrick, M. Lonergan, C. Collins, L. Daly. GP-delivered secondary prevention cardiovascular disease programme; early predictors of likelihood of patient non-adherence. European Journal of General Practice. 2010. 16:241-243 |  |  |  |  | Y |  |
| T. M. Beckie, J. W. Beckstead. Predicting cardiac rehabilitation attendance in a gender-tailored randomized clinical trial. Journal of cardiopulmonary rehabilitation and prevention. 2010. 30:147-156 |  |  | Y |  |  |  |
| C. Sherrington, S. R. Lord, C. M. Vogler, J. C. Close, K. Howard, C. M. Dean, L. Clemson, E. Barraclough, E. Ramsay, S. D. O'Rourke, R. G. Cumming. Minimising disability and falls in older people through a post-hospital exercise program: a protocol for a randomised controlled trial and economic evaluation. BMC geriatrics. 2009. 9:#pages# |  |  |  |  |  | Study protocol |
| H. Q. Nguyen, D. P. Gill, S. Wolpin, B. G. Steele, J. O. Benditt. Pilot study of a cell phone-based exercise persistence intervention post-rehabilitation for COPD. International journal of chronic obstructive pulmonary disease. 2009. 4:301-313 |  |  |  |  |  | No multivariable |
| R. N. Latka, M. Alvarez-Reeves, L. Cadmus, M. L. Irwin. Adherence to a randomized controlled trial of aerobic exercise in breast cancer survivors: the Yale exercise and survivorship study. Journal of cancer survivorship. 2009. 3:148-157 |  |  | Y |  |  |  |
| J. L. Huberty, J. Vener, N. Waltman, C. Ott, J. Twiss, G. Gross, R. McGuire, A. Dwyer. Development of an instrument to measure adherence to strength training in postmenopausal breast cancer survivors. Oncology Nursing Forum. 2009. 36:E266-73 |  |  | Y |  |  |  |
| R. L. Skolasky, E. J. Mackenzie, S. T. Wegener, L. H. Riley. Patient activation and adherence to physical therapy in persons undergoing spine surgery. Spine (03622436). 2008. 33:E784-91 |  |  | Y |  |  |  |
| A. Mayoux-Benhamou, J. S. Giraudet-Le Quintrec, P. Ravaud, K. Champion, E. Dernis, D. Zerkak, C. Roy, A. Kahan, M. Revel, M. Dougados. Influence of patient education on exercise compliance in rheumatoid arthritis: a prospective 12-month randomized controlled trial. The Journal of rheumatology. 2008. 35:216-23 |  |  | Y |  |  |  |
| P. L. Dobkin, R. Ionescu-Ittu, M. Abrahamowicz, M. Baron, S. Bernatsky, A. Sita. Predictors of adherence to an integrated multimodal program for fibromyalgia. Journal of Rheumatology. 2008. 35:2255-2264 |  |  | Y |  |  |  |
| K. S. Courneya, R. J. Segal, K. Gelmon, R. D. Reid, J. R. Mackey, C. M. Friedenreich, C. Proulx, K. Lane, A. B. Ladha, J. K. Vallance, D. C. McKenzie. Predictors of supervised exercise adherence during breast cancer chemotherapy. Medicine and science in sports and exercise. 2008. 40:1180-1187 |  |  | Y |  |  |  |
| K. S. Courneya, D. C. McKenzie, R. D. Reid, J. R. Mackey, K. Gelmon, C. M. Friedenreich, A. B. Ladha, C. Proulx, K. Lane, J. K. Vallance, R. J. Segal. Barriers to supervised exercise training in a randomized controlled trial of breast cancer patients receiving chemotherapy. Annals of behavioral medicine : a publication of the Society of Behavioral Medicine. 2008. 35:116-22 |  |  | Y |  |  |  |
| A. B. Moadel, C. Shah, J. Wylie-Rosett, M. S. Harris, S. R. Patel, C. B. Hall, J. A. Sparano. Randomized controlled trial of yoga among a multiethnic sample of breast cancer patients: effects on quality of life. Journal of clinical oncology. 2007. 25:4387-4395 |  |  | Y |  |  |  |
| D. Donesky-Cuenco, S. Janson, J. Neuhaus, T. B. Neilands, V. Carrieri-Kohlman. Adherence to a home-walking prescription in patients with chronic obstructive pulmonary disease. Heart & Lung. 2007. 36:348-363 |  | Y |  |  |  |  |
| D. L. Mori, S. Sogg, P. Guarino, J. Skinner, D. Williams, A. Barkhuizen, C. Engel, D. Clauw, S. Donta, P. Peduzzi. Predictors of exercise compliance in individuals with Gulf War veterans illnesses: Department of Veterans Affairs Cooperative Study 470. Military medicine. 2006. 171:917-23 |  |  | Y |  |  |  |
| H. M. McGee, F. Doyle, R. M. Conroy, D. De La Harpe, E. Shelley. Impact of briefly-assessed depression on secondary prevention outcomes after acute coronary syndrome: a one-year longitudinal survey. BMC health services research. 2006. 6:13 |  |  | Y |  |  |  |
| T. Y. Liu-Ambrose, K. M. Khan, J. J. Eng, S. R. Lord, B. Lentle, H. A. McKay. Both resistance and agility training reduce back pain and improve health-related quality of life in older women with low bone mass. Osteoporosis international. 2005. 16:1321-1329 |  |  | Y |  |  |  |
| K. Harkness, K. M. Smith, L. Taraba, C. L. MacKenzie, E. Gunn, H. M. Arthur. Effect of a postoperative telephone intervention on attendance at intake for cardiac rehabilitation after coronary artery bypass graft surgery. Heart & Lung. 2005. 34:179-186 |  |  | Y |  |  |  |
| K. A. Schutzer, B. S. Graves. Barriers and motivations to exercise in older adults. Preventive Medicine. 2004. 39:1056-1061 |  |  |  | Y |  |  |
| Anya Whitmarsh, Maria Koutantji, Kate Sidell. Illness perceptions, mood and coping in predicting attendance at cardiac rehabilitation. British Journal of Health Psychology. 2003. 8:209-221 |  |  | Y |  |  |  |
| D. D. Cuenco. Adherence to exercise in patients with chronic obstructive pulmonary disease. #journal#. 2003. Ph.D.:162 p-162 p |  |  |  |  |  | Dissertation |
| S. Herman, J. A. Blumenthal, M. Babyak, P. Khatri, W. E. Craighead, K. R. Krishnan, P. M. Doraiswamy. Exercise therapy for depression in middle-aged and older adults: predictors of early dropout and treatment failure. Health Psychology. 2002. 21:553-563 |  |  | Y |  |  |  |
| K. S. Courneya, C. M. Friedenreich, R. A. Sela, H. A. Quinney, R. E. Rhodes. Correlates of adherence and contamination in a randomized controlled trial of exercise in cancer survivors: an application of the theory of planned behavior and the five factor model of personality. Annals of behavioral medicine : a publication of the Society of Behavioral Medicine. 2002. 24:257-68 |  |  | Y |  |  |  |
| A. F. Cooper, G. Jackson, J. Weinman, R. Horne. Factors associated with cardiac rehabilitation attendance: a systematic review of the literature. Clinical Rehabilitation. 2002. 16:541-552 |  |  |  |  |  | Review |
| G. S. Bauldoff, L. A. Hoffman, T. G. Zullo, F. C. Sciurba. Exercise maintenance following pulmonary rehabilitation: effect of distractive stimuli. Chest. 2002. 122:948-54 |  | Y |  |  |  |  |
| N. T. James. Exercise compliance in older adults with arthritis. #journal#. 2001. Ph.D.:176 p-176 p |  |  |  |  |  | Dissertation |
| J. J. Carlson, G. J. Norman, D. L. Feltz, B. A. Franklin, J. A. Johnson, S. K. Locke. Self-efficacy, psychosocial factors, and exercise behavior in traditional versus modified cardiac rehabilitation. Journal of cardiopulmonary rehabilitation. 2001. 21:363-373 |  |  | Y |  |  |  |
| R. Campbell, M. Evans, M. Tucker, B. Quilty, P. Dieppe, J. L. Donovan. Why don't patients do their exercises? Understanding non-compliance with physiotherapy in patients with osteoarthritis of the knee. Journal of epidemiology and community health. 2001. 55:132-138 |  | Y |  |  |  |  |
| C. Chen, P. S. Neufeld, C. A. Feely, C. S. Skinner. Factors influencing compliance with home exercise programs among patients with upper-extremity impairment. American Journal of Occupational Therapy. 1999. 53:171-180 |  |  | Y |  |  |  |
| W. J. Rejeski, L. R. Brawley, W. Ettinger, T. Morgan, C. Thompson. Compliance to exercise therapy in older participants with knee osteoarthritis: implications for treating disability. Medicine and science in sports and exercise. 1997. 29:977-85 |  |  |  | Y |  | Wrong population |
| E. A. Hellman. Use of the Stages of Change in Exercise Adherence model among older adults with a cardiac diagnosis. Journal of Cardiopulmonary Rehabilitation. 1997. 17:145-155 | Y |  |  |  |  |  |
| J. Roomi, M. M. Johnson, K. Waters, A. Yohannes, A. Helm, M. J. Connolly. Respiratory rehabilitation, exercise capacity and quality of life in chronic airways disease in old age. Age & Ageing. 1996. 25:12-16 |  | Y |  |  |  |  |
| D. E. Sassi-Dambron, E. G. Eakin, A. L. Ries, R. M. Kaplan. The effects of compliance with exercise training on pulmonary rehabilitation. Rehabilitation Nursing Research. 1994. 3:3-10 |  |  | Y |  |  |  |
| M. A. Minor, J. D. Brown. Exercise maintenance of persons with arthritis after participation in a class experience. Health education quarterly. 1993. 20:83-95 |  |  | Y |  |  |  |
| A. A. E. Kartha. Factors influencing the adherence behaviors of elderly incontinent women. #journal#. 1989. PH.D.:216 p-216 p |  |  |  |  |  | Dissertation |
| P. Miller, R. Wikoff, M. McMahon, M. J. Garrett, K. Ringel. Influence of a nursing intervention on regimen adherence and societal adjustments postmyocardial infarction. Nursing research. 1988. 37:297-302 |  |  | Y |  |  |  |
| S. Banerjee, K. Manley, L. Thomas, B. Shaw, J. Saxton, R. Mills, M. Rochester. Preoperative exercise protocol to aid recovery of radical cystectomy: Results of a feasibility study. European Urology, Supplements. #year#. 12:125-6 |  |  |  |  |  | Abstract only |
| Williamson, Tamara M, Rouleau, Codie R, Aggarwal, Sandeep G, Arena, Ross, Campbell, Tavis S. Bridging the intention-behavior gap for cardiac rehabilitation participation: the role of perceived barriers. Disability & Rehabilitation. 2020///. 42:1284 |  |  | Y |  |  |  |
| Sun, Virginia, Raz, Dan J, Kim, Jae Y, Melstrom, Laleh, Hite, Sherry, Varatkar, Gouri, Fong, Yuman. Barriers and facilitators of adherence to a perioperative physical activity intervention for older adults with cancer and their family caregivers. Journal of geriatric oncology. #year#. 11:256 |  |  | Y |  |  |  |
| Sr, P A A, DeFeis, B, De Wit, L, O'Shea, D, Mejia, A, Chandler, M, Locke, D E C, Fields, J, Phatak, V, Dean, P M, Crook, J, Smith, G. Functional ability is associated with higher adherence to behavioral interventions in mild cognitive impairment. The Clinical neuropsychologist. 2020///. 34:937 |  |  |  | Y |  |  |
| Schreiner, Nathanial, DiGennaro, Sarah, Harwell, Carla, Burant, Christopher, Daly, Barbara, Douglas, Sara. Treatment burden as a predictor of self-management adherence within the primary care population. Applied Nursing Research. 2020///. 54:N.PAG |  |  | Y |  |  |  |
| Reed, Phil, Whittall, C Mair, Osborne, Lisa A, Emery, Simon. Impact of Strength and Nature of Patient Health Values on Compliance and Outcomes for Physiotherapy Treatment for Pelvic Floor Dysfunction. Urology. #year#. 136:95 |  |  | Y |  |  |  |
| Pastor-Mira, Maria-Angeles, Lopez-Roig, Sofia, Penacoba, Cecilia, Sanz-Banos, Yolanda, Lledo, Ana, Velasco, Lilian. Predicting walking as exercise in women with fibromyalgia from the perspective of the theory of planned behavior. Women & health. #year#. 60:412 |  |  | Y |  |  |  |
| O'Bertos, Shea E, Holmberg, Diane, Shields, Christopher A, Matheson, Lauren P. Partners' Attachment Styles and Overprotective Support as Predictors of Patient Outcomes in Cardiac Rehabilitation. Rehabilitation Psychology. 2020///. 65:258 |  |  |  |  |  | No multivariable |
| Northgraves, Matthew J, Arunachalam, Lakshmanan, Madden, Leigh A, Marshall, Philip, Hartley, John E, MacFie, John, Vince, Rebecca V. Feasibility of a novel exercise prehabilitation programme in patients scheduled for elective colorectal surgery: a feasibility randomised controlled trial. Supportive Care in Cancer. 2020///. 28:3197 |  | Y |  |  |  |  |
| Liu, M, Miyawaki, C E. What Types of Physical Function Predict Program Adherence in Older Adults?. Rehabilitation nursing : the official journal of the Association of Rehabilitation Nurses. 2020///. 45:279 |  |  |  | Y |  |  |
| Jankowska-Polanska, Beata, Swiatoniowska-Lonc, Natalia, Slawuta, Agnieszka, Krowczynska, Dorota, Dudek, Krzysztof, Mazur, Grzegorz. Patient-Reported Compliance in older age patients with chronic heart failure. PloS one. #year#. 15:e0231076 | Y |  |  |  |  |  |
| Hyoung Sook, Park, Yun Seo, Jung, Young, Kim, Jae Hyun, Hwang. A Study on Breast Cancer Patients' Commitment to a Plan for Exercise based on Health Beliefs. Journal of Korean Academy of Fundamentals of Nursing. 2020///. 27:64 |  |  |  |  |  | Korean |
| Claes, Jomme, Cornelissen, Véronique, McDermott, Clare, Moyna, Niall, Pattyn, Nele, Cornelis, Nils, Gallagher, Anne, McCormack, Ciara, Newton, Helen, Gillain, Alexandra, Budts, Werner, Goetschalckx, Kaatje, Woods, Catherine, Moran, Kieran, Buys, Roselien. Feasibility, Acceptability, and Clinical Effectiveness of a Technology-Enabled Cardiac Rehabilitation Platform (Physical Activity Toward Health-I): Randomized Controlled Trial. Journal of Medical Internet Research. 2020///. 22:N.PAG |  |  | Y |  |  |  |
| Blackwell, J E M, Doleman, B, Boereboom, C L, Morton, A, Williams, S, Atherton, P, Smith, K, Williams, J P, Phillips, B E, Lund, J N. High-intensity interval training produces a significant improvement in fitness in less than 31 days before surgery for urological cancer: a randomised control trial. Prostate cancer and prostatic diseases. #year#. #volume#:#pages# |  | Y |  |  |  |  |
| Albergoni, A, Hettinga, F J, Stut, W, Sartor, F. Factors influencing walking and exercise adherence in healthy older adults using monitoring and interfacing technology: Preliminary evidence. International Journal of Environmental Research and Public Health. 2020///. 17:1 |  |  |  |  |  | No multivariable |
| Sørensen, Dorthe, Christensen, Marie Ernst. Behavioural modes of adherence to inspiratory muscle training in people with chronic obstructive pulmonary disease: a grounded theory study. Disability & Rehabilitation. 2019///. 41:1071 |  | Y |  |  |  |  |
| Ramkumar, P N, Haeberle, H S, Ramanathan, D, Cantrell, W A, Navarro, S M, Mont, M A, Bloomfield, M, Patterson, B M. Remote Patient Monitoring Using Mobile Health for Total Knee Arthroplasty: Validation of a Wearable and Machine Learning-Based Surveillance Platform. Journal of Arthroplasty. 2019///. 34:2253 |  | Y |  |  |  |  |
| Piva, Sara R, Khoja, Samannaaz S, Toledo, Frederico G S, Chester‐Wasko, Mary, Fitzgerald, G Kelley, Goodpaster, Bret H, Smith, Clair N, Delitto, Anthony, Chester-Wasko, Mary. Neuromuscular Electrical Stimulation Compared to Volitional Exercise for Improving Muscle Function in Rheumatoid Arthritis: A Randomized Pilot Study. Arthritis Care & Research. 2019///. 71:352 |  | Y |  |  |  |  |
| Northey, Joseph M, Pumpa, Kate L, Quinlan, Clare, Ikin, Ashley, Toohey, Kellie, Smee, Disa J, Rattray, Ben. Cognition in breast cancer survivors: A pilot study of interval and continuous exercise. Journal of Science & Medicine in Sport. 2019///. 22:580 |  | Y |  |  |  |  |
| Niedermann, Karin, Nast, Irina, Ciurea, Adrian, Vliet Vlieland, Thea, Bodegom‐Vos, Leti, van Bodegom-Vos, Leti. Barriers and Facilitators of Vigorous Cardiorespiratory Training in Axial Spondyloarthritis: Surveys Among Patients, Physiotherapists, and Rheumatologists. Arthritis Care & Research. 2019///. 71:839 | Y |  |  |  |  |  |
| Nct. Virtual Reality and Video Games in Cardiac Rehabilitation Programs. Virtual Reality and Video Games in Cardiac Rehabilitation Programs. A Randomized Controlled Trial. 2019///. #volume#:#pages# | Y |  |  |  |  |  |
| Nct. Evaluating Effect of Cognitive Game Based Treadmill Exercise Program in Parkinson Disease. Evaluating the Use of a Cognitive Game-Based Treadmill Exercise Program to Improve Balance and Gait in People With Parkinson Disease: a Feasibility Randomized Controlled Trial. 2019///. #volume#:#pages# | Y |  |  |  |  |  |
| Myers, Jamie S, Mitchell, Melissa, Krigel, Susan, Steinhoff, Andreanna, Boyce-White, Alyssa, Van Goethem, Karla, Valla, Mary, Dai, Junqiang, He, Jianghua, Liu, Wen, Sereika, Susan M, Bender, Catherine M. Qigong intervention for breast cancer survivors with complaints of decreased cognitive function. Supportive Care in Cancer. 2019///. 27:1395 |  | Y |  |  |  |  |
| Milot Mh, Leonard G Corriveau H Desrosiers J. Using the Borg rating of perceived exertion scale to grade the intensity of a functional training program of the affected upper limb after a stroke: a feasibility study. Clinical interventions in aging. 2019///. 14:9 |  | Y |  |  |  |  |
| Lacey, Judith, Lomax, Anna J, McNeil, Catriona, Marthick, Michael, Levy, David, Kao, Steven, Nielsen, Theresa, Dhillon, Haryana M. A supportive care intervention for people with metastatic melanoma being treated with immunotherapy: a pilot study assessing feasibility, perceived benefit, and acceptability. Supportive Care in Cancer. 2019///. 27:1497 |  | Y |  |  |  |  |
| Klinovszky, A, Marton Kiss, I, Papp-Zipernovszky, O, Lengyel, C, Buzas, N. Associations of different adherences in patients with type 2 diabetes mellitus. Patient Preference and Adherence. 2019///. 13:395 |  |  | Y |  |  |  |
| Gibbs, J C, Carvalho, L P, Marcangeli, V, El Hajj Boutros, G, Dulac, M C, Aubertin-Leheudre, M. Effect of high-intensity interval training on peripheral quantitative computed tomography measures of quadriceps muscle and adipose tissue properties in obese, osteopenic older women. Journal of Bone and Mineral Research. 2019///. 34:156 |  |  |  |  |  | Abstract only |
| Ge, C, Ma, J, Xu, Y, Shi, Y J, Zhao, C H, Gao, L, Bai, J, Wang, Y, Sun, Z J, Guo, J, Chen, Y D. Predictors of adherence to home-based cardiac rehabilitation program among coronary artery disease outpatients in China. Journal of Geriatric Cardiology. 2019///. 16:749 |  |  | Y |  |  |  |
| Gaalema, Diann E, Dube, Sarahjane, Potter, Alexandra, Elliott, Rebecca J, Mahoney, Katharine, Sigmon, Stacey C, Higgins, Stephen T, Ades, Philip A. The effect of executive function on adherence with a cardiac secondary prevention program and its interaction with an incentive-based intervention. Preventive medicine. #year#. 128:105865 |  |  | Y |  |  |  |
| Foucaut, Aude-Marie, Morelle, Magali, Kempf-Lépine, Anne-Sophie, Baudinet, Cédric, Meyrand, Renaud, Guillemaut, Séverine, Metzger, Séverine, Bourne-Branchu, Valérie, Grinand, Elodie, Chabaud, Sylvie, Pérol, David, Carretier, Julien, Berthouze, Sophie E, Reynes, Eric, Perrier, Lionel, Rebattu, Paul, Heudel, Pierre-Etienne, Bachelot, Thomas, Bachmann, Patrick, Fervers, Béatrice. Feasibility of an exercise and nutritional intervention for weight management during adjuvant treatment for localized breast cancer: the PASAPAS randomized controlled trial. Supportive Care in Cancer. 2019///. 27:3449 |  |  | Y |  |  |  |
| Fernández-González, Pilar, Carratalá-Tejada, María, Monge-Pereira, Esther, Collado-Vázquez, Susana, Sánchez-Herrera Baeza, Patricia, Cuesta-Gómez, Alicia, Oña-Simbaña, Edwin Daniel, Jardón-Huete, Alberto, Molina-Rueda, Francisco, Balaguer-Bernaldo de Quirós, Carlos, Miangolarra-Page, Juan Carlos, Cano-de la Cuerda, Roberto. Leap motion controlled video game-based therapy for upper limb rehabilitation in patients with Parkinson's disease: a feasibility study. Journal of NeuroEngineering & Rehabilitation (JNER). 2019///. 16:N.PAG |  | Y |  |  |  |  |
| Blackwell, J, Boereboom, C, Doleman, B, Phillips, B, Williams, J, Lund, J. High intensity interval training is a safe and effective way to improve fitness before surgery for cancer: A randomised control trial. British Journal of Surgery. 2019///. 106:39 |  | Y |  |  |  |  |
| Adsett, Julie A, Morris, Norman R, Mudge, Alison M. Predictors of Exercise Training and Physical Activity Adherence in People Recently Hospitalized With Heart Failure: A BRIEF REPORT. Journal of cardiopulmonary rehabilitation and prevention. #year#. 39:E12 |  |  | Y |  |  |  |
| Aalami, O, Savage, D, Rens, N, Meng, E, Triggs, D. PC086. Mobile Engagement for Walking in Patients With Claudication. Journal of Vascular Surgery. 2019///. 69:e230 |  |  |  |  |  | Abstract only |
| Jones, M C, Smith, K, Herber, O, White, M, Steele, F, Johnston, D W. Intention, beliefs and mood assessed using electronic diaries predicts attendance at cardiac rehabilitation: An observational study. International journal of nursing studies. 2018///. 88:143 |  |  | Y |  |  |  |
| Duscha, Brian D, Piner, Lucy W, Patel, Mahesh P, Craig, Karen P, Brady, Morgan, IiimcGarrah, Robert W, Chen, Connie, Kraus, William E, McGarrah 3rd, Robert W. Effects of a 12-week mHealth program on peak VO2 and physical activity patterns after completing cardiac rehabilitation: A randomized controlled trial. American Heart Journal. 2018///. 199:105 |  |  | Y |  |  |  |
