# Supplementary material for: Predictors of adherence to prescribed exercise programs for older adults with medical or surgical indications for exercise: a systematic review
Source: Syst Rev. 2022 Apr 29;11:80. doi: 10.1186/s13643-022-01966-9 (PMC9052492; doi:10.1186/s13643-022-01966-9)
Supplement: Supplementary file 4 — Additional file 4: Supplementary Table S4. Predictors of Exercise Adherence for Cardiac Rehabilitation. [file 13643_2022_1966_MOESM4_ESM.docx]

**Supplementary Table S4. Predictors of Exercise Adherence for Cardiac Rehabilitation**

| Study | Predictors | Cluster | Effect size (95% CI) | Direction |
| --- | --- | --- | --- | --- |
| Casey et al. (2008) | Age | D | OR 1.04 (1.03–1.06), p < 0.01 | + |
|  | Employed (vs not employed/retired) | D | OR 1.37 (0.78–2.40) | 0 |
|  | Male (vs female) | D | OR 1.34 (0.86–2.06) | 0 |
|  | BMI | C | OR 0.99 (0.95–1.02) | 0 |
|  | Depression (BDI) | P | OR 0.96 (0.93–0.99), p < 0.01 | - |
| Tooth et al. (1992) | Scale of Status and Prestige score (high score = lower social standing) | D | p < 0.05 | - |
|  | Age | D | p > 0.05 | 0 |
|  | Education | D | p > 0.05 | 0 |
|  | CHD duration | S | p > 0.05 | 0 |
|  | Time since MI | S | p > 0.05 | 0 |
|  | Cigarettes (packyears) | C | p > 0.05 | 0 |
|  | Hypertension | C | p > 0.05 | 0 |
|  | Obesity (BMI) | C | p > 0.05 | 0 |
|  | Cholesterol | C | p > 0.05 | 0 |
|  | Expectations (high score = optimistic) | P | p < 0.05 | + |
|  | Psychological status (POMS) | P | p > 0.05 | 0 |
|  | Previous Exercise (MET hours/week) | O | p > 0.05 | 0 |
| Gallagher et al. (2003) | Unemployed or retired | D | OR 0.20 (0.07-0.58), p < 0.01 | - |
|  | Age 55–70 (vs >70) | D | OR 1.72 (1.10 -2.70), p < 0.01 | + |
|  | CABG diagnosis (vs MI) | S | OR 6.82 (1.84-25.21), p < 0.01 | + |
|  | Perceived control | P | OR 0.92 (0.85-1.00), p < 0.01 | - |
|  | Personal stressful event | P | OR 0.21 (0.06-0.73), p < 0.01 | - |
| Pakzad et al. (2013) | Identity | D | β = 0.45, t = 0.54, b = 0.11, p > 0.01 | 0 |
|  | HDL cholesterol | C | β = 18.53, t = 2.74, b = 0.41, p < 0.01 , F = 9.15 | + |
|  | Anxiety | P | β = 0.91, t = 2.73, b = 0.41, p < 0.01, F = 9.36 | + |
|  | Consequences | P | β = -0.37, t = -0.73, b = -0.16, p > 0.01 | 0 |
|  | Chronology (acute/chronic) | P | β = 0.23, t = 0.61, b = 0.11, p > 0.01 | 0 |
|  | Treatment control | P | β = -0.31, t = -0.36, b = - 0.08, p > 0.01 | 0 |
|  | Personal control | P | β =0.18, t = 0.81, b = 0.06, p > 0.01 | 0 |
| Ades et al. (1992) | Strength of physician recommendation | O | p < 0.05 | + |
|  | Commute time | E | p < 0.05 | - |
|  | Patient "denying" severity of illness | O | p < 0.05 | - |
|  | Presence of depression before hospitalization | O | p < 0.05 | - |
| van Montfort et al. (2016) | Female (vs male) | D | β = -0.15 (p=0.271); β = -0.07 (p=0.264) | 0 |
|  | Age | D | Model 1: β = -0.13 (p=0.019); Model 2: β = -0.16 (p=0.010) | - |
|  | Optimism (LOT-R) | P | Model 1: β = 0.23 (p=0.0001); Model 2: β = 0.23 (p=0.0001) | + |
|  | Acute PCI | S | Model 1: β = 0.01 (p=0.960); Model 2: β = 0.00 (p=0.955) | 0 |
|  | Cardiac history | C | Model 1: β = 0.02 (p=0.643); Model 2: β = 0.03 (p=0.586) | 0 |
|  | Depression (PHQ-9) | P | Model 1: β = -0.08 (p=0.139) | 0 |
|  | Anxiety (GAD-7) | P | Model 2: β = -0.16 (p=0.014) | - |

CI = confidence interval; D = demographic factors; C = comorbidities; P = psychological factors; S = medical condition severity; O = other factors; E = exercise program factors; BMI = body mass index; BDI = Beck Depression Inventory; POMS = Profile of Mood States; CHD = coronary heart disease; MI = myocardial infarction; CABG = coronary artery bypass graft; PCI = percutaneous coronary intervention; LOT-R = Revised Life Orientation Test; PHQ-9 = Patient Health Questionnaire; GAD-7 = Generalized Anxiety Disorder scale
