# Supplementary material for: Predictors of adherence to prescribed exercise programs for older adults with medical or surgical indications for exercise: a systematic review
Source: Syst Rev. 2022 Apr 29;11:80. doi: 10.1186/s13643-022-01966-9 (PMC9052492; doi:10.1186/s13643-022-01966-9)
Supplement: Supplementary file 5 — Additional file 5: Supplementary Table S5. Predictors of Exercise Adherence for Pulmonary Rehabilitation. [file 13643_2022_1966_MOESM5_ESM.docx]

**Supplementary Table S5. Predictors of Exercise Adherence for Pulmonary Rehabilitation**

| Study | Predictors | Cluster | Effect size (95% CI) | Direction |
| --- | --- | --- | --- | --- |
| Fan et al. (2008) | Age (per 1 year change) | D | OR 1.01 (0.99–1.04) | 0 |
|  | Distance to rehabilitation in miles (in quartiles) reference: < 6 miles | E | OR 1.0 |  |
|  | 6-14.9 miles | E | OR 0.86 (0.55-1.36) | 0 |
|  | 15-35.9 miles | E | OR 0.91 (0.57-1.44) | 0 |
|  | > 36 miles | E | OR 0.49 (0.31-0.75) | - |
|  | Female (vs male) | D | OR 0.87 (0.62-1.21) | 0 |
|  | Education reference: < high school | D | OR 1.0 |  |
|  | High school | D | OR 1.58 (1.05-2.38) | + |
|  | Some college | D | OR 1.66 (1.10-2.51) | + |
|  | > College | D | OR 2.21 (1.27-3.85) | + |
|  | Daily alcohol use | C | OR 1.33 (0.82-2.17) | 0 |
|  | Charlson Cormorbidity score  reference: 0 | C | OR 1.0 |  |
|  | > 1 | C | OR 1.13 (0.82-1.56) | 0 |
|  | BMI | C | OR 1.00 (0.96-1.04) | 0 |
|  | FEV1 reference < 20% | S | OR 1.0 |  |
|  | FEV1 ≥20% | S | OR 1.67 (1.09–2.55) | + |
|  | Total SOBQ score | S | OR 1.04 (0.99-1.09) | 0 |
|  | Distance walked in 6 mins | S | OR 1.01 (0.91 -1.12) | 0 |
|  | Randomized to LVRS were more likely to be adherent | E | OR 2.43 (1.77– 3.35) | + |
|  | STAI reference < 36 | P | OR 1.0 |  |
|  | STAI ≥ 36 | P | OR 0.65 (0.47-0.91) | - |
|  | BDI reference < 5 | P | OR 1.0 |  |
|  | BDI ≥ 5 | P | OR 0.55 (0.34-0.87) | - |
| Brown et al. (2016) | FEV1 | s | OR 0.71 (0.38-1.34) | 0 |
|  | FVC | S | OR 0.86 (0.58-1.29) | 0 |
|  | SF-36 | S | OR 1.01 (0.99–1.02) | 0 |
|  | Smoking at enrollment | S | OR 0.38 (0.16–0.90), p = 0.02 | - |
|  | Depression (BDI) | P | OR 1.01 (0.97-1.05) | 0 |
| Rizk et al (2015) | Lower in IT compared to CTHI and CTVT | E | F = 6.69, p < 0.01 | - |
| Hogg et al. (2012) | Deprivation quintile (IMD score) reference: IMD 6.86-28.1 | D | OR 1.0 |  |
|  | IMD 28.11–35.02 | D | OR 0.72 (0.43-1.2) | 0 |
|  | IMD 35.03–39.57 | D | OR 1.0 (0.59–1.7) | 0 |
|  | IMD 39.58–43.85 | D | OR 0.56 (0.33–0.94), p < 0.05 | - |
|  | IMD 43.86–60.41 | D | OR 0.57 (0.34–0.85), p < 0.05 | - |
|  | MRC dyspnoea score reference MRC 1 or 2 | S | OR 1.0 |  |
|  | MRC 3 | S | OR 0.88 (0.55–1.41) | 0 |
|  | MRC 4 | S | OR 0.61 (0.37–0.97), p < 0.05 | - |
|  | MRC 5 | S | OR 0.39 (0.16–0.93), p < 0.05 | - |
|  | Depression score (HADS) reference "not depressed" 0-7 | P | OR 1.0 |  |
|  | Risk of depression 8-10 | P | 0.77(0.51–1.18) | 0 |
|  | Depressed 11 | P | 0.56 (0.37–0.85), p < 0.05 | - |
|  | Source of referral reference: Consultant respiratory physician | O | OR 1.0 |  |
|  | In-patient COPD multidisciplinary team | O | 0.87 (0.48–1.57) | 0 |
|  | Hospital physiotherapist | O | 0.55 (0.27 - 1.15) | 0 |
|  | General practitioner referral | O | 0.42 (0.24–0.74), p < 0.05 | - |
|  | Practice nurse referral | O | 0.89 (0.54 - 1.48) | 0 |
|  | Community COPD clinic referral | O | 0.82 (0.49 - 1.35) | 0 |
|  | Other | O | 0.55 (0.15 - 2.07) | 0 |
| Selzler et al. (2016) | Age | D | β = 0.22, p = 0.12, r = 0.10, partial r = 0 .19 | 0 |
|  | Baseline SGRQ | S | β = -0.15, p = 0.23, r = -0.22, partial r = -0.15 | 0 |
|  | Smoking history | C | β = -0.07, p= 0.57, r = -0.15, partial r = -0 .07 | 0 |
|  | Task self-efficacy | P | β = 0.32, p = 0.04, r =0.23, partial r = 0.26 | + |
|  | Coping self-efficacy (confidence for exercising when you feel discomfort) | P | β = -0.16, p = 0.26, r =0.03, partial r = -0.10 | 0 |
|  | Scheduling self-efficacy (confidence for arranging schedule to include regular exercise) | P | β = 0.12, p = 0.42, r = 0.29, partial r = 0.10 | 0 |
| Selzler et al. (2012) | Age | D | Discriminant function coefficient = 0.65 | + |
|  | General health | S | Discriminant function coefficient = 0.34 | + |
|  | Physical functioning | S | Discriminant function coefficient = 0.41 | + |
|  | Total SGRQ | S | Discriminant function coefficient = -0.44 | - |
|  | Smoker | C | Discriminant function coefficient = 0.44 | + |
|  | Bodily pain | C | Discriminant function coefficient = 0.63 | + |
|  | Vitality | C | Discriminant function coefficient = 0.52 | + |
|  | Social functioning | P | Discriminant function coefficient = 0.56 | + |
|  | Mental health | P | Discriminant function coefficient = 0.45 | + |
|  | Role emotional | P | Discriminant function coefficient = 0.35 | + |
| Covey et al. (2014) | Resistance training (RT) then aerobic training (AT) vs Combined RT+AT vs AT (attendance between three groups) | E | p = 0.67 | 0 |

CI = confidence interval; D = demographic factors; C = comorbidities; P = psychological factors; S = medical condition severity; O = other factors; E = exercise program factors; BMI = body mass index; FEV1 = forced expiratory volume in 1 second; SOBQ = shortness of breath questionnaire; LVRS = lung volume reduction surgery; STAI = State-Trait Anxiety Inventory; BDI = Beck Depression Inventory; FVC = forced vital capacity; SF-36 = 36-Item Short Form Survey; IT = interval training; CTHI = continuous training at high intensity; CTVT = continuous training at ventilatory threshold; IMD = Index of Multiple Deprivation Score; MRC = Medical Research Council dyspnoea scale; HADS = Hospital Anxiety and Depression Scale; COPD = chronic obstructive pulmonary disease; SGRQ = St. Georges Respiratory Questionnaire;
