# Supplementary material for: Predictors of adherence to prescribed exercise programs for older adults with medical or surgical indications for exercise: a systematic review
Source: Syst Rev. 2022 Apr 29;11:80. doi: 10.1186/s13643-022-01966-9 (PMC9052492; doi:10.1186/s13643-022-01966-9)
Supplement: Supplementary file 6 — Additional file 6: Supplementary Table S6. Predictors of Exercise Adherence for Other Exercise Programs. [file 13643_2022_1966_MOESM6_ESM.docx]

**Supplementary Table S6. Predictors of Exercise Adherence for Other Exercise Programs**

| Study | Predictors | Cluster | Effect size (95% CI) | Direction |
| --- | --- | --- | --- | --- |
| Pandey et al (2017) | Type of exercise program. moderate-intensity continuous exercise training control, burst exercise intervention | E | p < 0.01 | + |
| Aherne et al. (2017) | Non-active smoker | C | p < 0.01 | + |
|  | Chronic obstructive pulmonary disease | C | N/A | 0 |
|  | Ischemic heart disease | C | N/A | 0 |
|  | Chronic kidney disease | C | N/A | 0 |
|  | Diabetes mellitus | C | N/A | 0 |
|  | Hypertension | C | N/A | 0 |
|  | Hypercholesterolemia | C | N/A | 0 |
| Craike et al. (2016) | Level of education | D | p > 0.10 | 0 |
|  | Medical severity | D |  |  |
|  | Hormonal symptoms | S | B = −0.483 (-0.976-0.009), p = 0.05 | - |
|  | Fatigue | S | p > 0.10 | 0 |
|  | Role functioning | P | B = 0.309 (0.051-0.568), p = 0.02 | + |
|  | Sexual activity | O | p > 0.10 | 0 |
| Tiedemann et al. (2012) | Conditions/symptoms (total number) | S | Coefficient 1.99 (-1.02-5.00), p = 0.19 | 0 |
|  | Fear of falling (yes/no) | O | Coefficient 7.50 (-4.93-19.93), p = 0.23 | 0 |
|  | Choice stepping reaction time (sec) | O | Coefficient 0.20 (−0.004-0.40), p = 0.06 | 0 |
| Messer et al. (2007) | Task SE summary scores predict adherence over time | P | β = 0.51, SE (β) = 0.23, p < 0.05 | + |
|  | Regulatory SE summary scores predict adherence over time | P | β = 0.56, SE (β) = 0.19, p < 0.01 | + |
|  | Knowledge SE | P | β = -0.20, SE (β) = 0.22, p > 0.05 | 0 |
| Cox et al. (2013) | Injury | C | 51.73 (45.00-58.40)% reduction in TAdh, p < 0.01 | - |
|  | Higher baseline BMI | C | 1.48 (0.33-2.63)% reduction in TAdh, p < 0.05 | - |
|  | Cognition (MMSE) | C | 4.46 (1.20-7.71)% reduction in TAdh, p < 0.01 | - |
|  | Higher baseline SE | P | 1.21 (0.22-2.0)% increase in TAdh, p < 0.01 | + |
| Mudge et al. (2013) | Being retired | D | RR 2.1 (1.1-2.5), p = 0.02 | + |
|  | Age (<65 vs 65+) | D | N/A not a predictor | 0 |
|  | Sex | D | N/A not a predictor | 0 |
|  | Living alone vs living with family/others | D | N/A not a predictor | 0 |
|  | Initial program vs maintenance program | E | N/A not a predictor | 0 |
|  | Rehabilitation type (cardiac, heart failure, pulmonary) | E | N/A not a predictor | 0 |
|  | Other community exercise classes vs No community exercise classes | O | N/A not a predictor | 0 |
|  | Confident in Heartmoves vs Not confident in Heartmoves | O | N/A not a predictor | 0 |
|  | Exercising 5+ days per week | O | RR 1.5 (1.0-1.8), p = 0.03 | + |
| Pickering et al. (2013) | Gender male (vs female) | D | RR 1.15 (0.98-1.36) p = 0.08 | 0 |
|  | Living status: Alone | D | RR 1.00, p = 0.70 |  |
|  | Partner | D | RR 1.05 (0.87-1.29) | 0 |
|  | Family/friend | D | RR 0.90 (0.68-1.21) | 0 |
|  | Other | D | RR 1.02 (0.51-2.04) | 0 |
|  | Age multiplicative decrease per 10 years | D | RR 0.90 (0.83-98), p = 0.01 | - |
|  | Year since PD diagnosis multiplicative decrease per 10 years | S | RR 0.95 (0.82-1.09), p = 0.47 | 0 |
|  | Self-assessment disability scale multiplicative decrease per 10 points (people with poorer physical state) | S | RR 0.91 (0.86-0.96), p < 0.01 | - |
|  | UPDRS motor exam multiplicative decrease per 10 points | S | RR 0.90 (0.81, 1.01), p = 0.0766 | 0 |
|  | Berg balance test multiplicative increase per 10 points | S | RR 1.14 (1.05, 1.24), p = 0.0018 | + |
|  | Functional reach multiplicative increase per 10 cm | S | RR 1.14 (1.01, 1.28), p = 0.0324 | + |
|  | Mental health problem | P | RR 0.80 (0.66-0.99), p = 0.0363 | - |
|  | EQ-5D No pain or discomfort (reference) | C | RR 1.00 |  |
|  | EQ-5D Moderate pain or discomfort | C | RR 0.90 (0.75, 1.09) | 0 |
|  | EQ-5D Extreme pain or discomfort | C | RR 0.64 (0.47, 0.87) | - |
|  | EQ-5D Not anxious or depressed (reference) | P | RR 1.00 |  |
|  | EQ-5D Moderately anxious or depressed | P | RR 0.84 (0.72, 0.97) | - |
|  | EQ-5D Extremely anxious or depressed | P | RR 0.47 (0.33, 0.68) | - |
|  | EQ-5D state of health thermometer multiplicative increase per 10 points | C | RR 1.05 (1.01, 1.11), p = 0.0190 | + |
| Jensen et al. (2016) | Gender male (vs female) | D | OR 1.24 (0.19-8.0), p=0.81 | 0 |
|  | Comorbidity Index > 2 (vs ≤2) | C | OR 0.94 (0.19-4.63), p=0.92 | 0 |
|  | Age ≥ 70 (vs < 70) | D | OR 4.11 (0.24-18.69), p=0.35 | 0 |
| Karssemeijer et al. (2019) | Exergame training group (EG) vs aerobic training group (AG) adherence | E | Mean difference 6.85 (-0.09, 13.69), p=0.05 | + |
|  |  |  |  |  |

CI = confidence interval; D = demographic factors; C = comorbidities; P = psychological factors; S = medical condition severity; O = other factors; E = exercise program factors; SE = self-efficacy; TAdh = total adherence; MMSE = Mini Mental State Examination; UPDRS = Unified Parkinson’s Disease Rating Scale
