# Supplementary material for: Predictors of adherence to prescribed exercise programs for older adults with medical or surgical indications for exercise: a systematic review
Source: Syst Rev. 2022 Apr 29;11:80. doi: 10.1186/s13643-022-01966-9 (PMC9052492; doi:10.1186/s13643-022-01966-9)
Supplement: Supplementary file 7 — Additional file 7: Supplementary Table S7. Risk of Bias Assessments for Observational Studies (QUIPS tool). [file 13643_2022_1966_MOESM7_ESM.docx]

**Supplementary Table S7. Risk of Bias Assessments for Observational Studies (QUIPS tool)**

| Author (Year) | Study Participation | Study Attrition | Prognostic Factor Measurement | Outcome Measurement | Study Confounding | Statistical Analysis and Reporting |
| --- | --- | --- | --- | --- | --- | --- |
| Ades et al. (1992) |  |  |  |  |  |  |
| Aherne et al. (2017) |  |  |  |  |  |  |
| Brown et al. (2016) |  |  |  |  |  |  |
| Casey et al. (2008) |  |  |  |  |  |  |
| Cox et al. (2013) |  |  |  |  |  |  |
| Craike et al. (2016) |  |  |  |  |  |  |
| Fan et al. (2008) |  |  |  |  |  |  |
| Gallagher et al. (2003) |  |  |  |  |  |  |
| Hogg et al. (2012) |  |  |  |  |  |  |
| Jensen et al. (2016) |  |  |  |  |  |  |
| Messer et al. (2007) |  |  |  |  |  |  |
| Mudge et al. (2013) |  |  |  |  |  |  |
| Pakzad et al. (2013) |  |  |  |  |  |  |
| Pickering et al. (2013) |  |  |  |  |  |  |
| Selzler et al. (2012) |  |  |  |  |  |  |
| Selzler et al. (2016) |  |  |  |  |  |  |
| Tiedemann et al. (2012) |  |  |  |  |  |  |
| Tooth et al. (1992) |  |  |  |  |  |  |
| van Montfort et al. (2016) |  |  |  |  |  |  |
|  |  |  |  |  |  |  |
|  | Low |  | Moderate |  |  | High |
