# Supplementary material for: Predictors of adherence to prescribed exercise programs for older adults with medical or surgical indications for exercise: a systematic review
Source: Syst Rev. 2022 Apr 29;11:80. doi: 10.1186/s13643-022-01966-9 (PMC9052492; doi:10.1186/s13643-022-01966-9)
Supplement: Supplementary file 8 — Additional file 8: Supplementary Table S8. Risk of Bias Assessments for Randomized Controlled Trials (Cochrane Risk of Bias tool). [file 13643_2022_1966_MOESM8_ESM.docx]

**Supplementary Table S8. Risk of Bias Assessments for Randomized Controlled Trials (Cochrane Risk of Bias tool)**

| Author (Year) | Random sequence generation | Allocation concealment | Blinding of participants and personnel | Blinding of outcome assessment | Incomplete outcome data | Selective reporting | Other bias |
| --- | --- | --- | --- | --- | --- | --- | --- |
| Covey et al. (2014) |  | * |  |  |  |  |  |
| Karssemeijer et al. (2019) | * | * |  |  |  |  |  |
| Pandey et al. (2017) | * | * |  | * |  |  |  |
| Rizk et al. (2015) | * | * |  | * |  |  |  |
|  |  |  |  |  |  |  |  |
|  | Low |  | Moderate |  |  | High | * Unclear |
